# Fucoxanthin and Its Variants from Seaweeds of the Arabian Gulf: Insight into Bioactivity, UV-Protective Properties, Stability and Cosmetic Potential

**DOI:** 10.3390/molecules31213878

**Published:** 2026-10-27

**Authors:** Sabeena Farvin Koduvayur Habeebullah, Surendraraj Alagarsamy, Salim Ok, Bernd Diehl, Mustafa Al-Shamali, Jessy Sebastian, Saja Fakhraldeen, Aws Al Ghunaim, Turki Al Said, Faiza Al-Yamani

**Affiliations:** 1 Coastal and Marine Resources Program, Environment and Life Sciences Research Center, Kuwait Institute for Scientific Research, P.O. Box 1638, Salmiya 22017, Kuwait; salagarsamy@kisr.edu.kw (S.A.); jsebastian@kisr.edu.kw (J.S.); sfakhraldeen@kisr.edu.kw (S.F.); aghunaim@kisr.edu.kw (A.A.G.); trsaid@kisr.edu.kw (T.A.S.); faizayamani@gmail.com (F.A.-Y.); 2 Petroleum Research Center, Kuwait Institute for Scientific Research, Safat 13109, Kuwait; saok@kisr.edu.kw (S.O.); mshamali@kisr.edu.kw (M.A.-S.); 3 Spectral Service AG, Emil-Hoffmann-Str. 33, 50996 Cologne, Germany; bernd.diehl@spectralservice.de

**Keywords:** fucoxanthin, UV-protection, antioxidant, anti-wrinkle, skin-whitening

## Abstract

The pigment profile of seaweeds thriving in the Arabian Gulf, characterized by high temperature, salinity, and high UV index, showed a high proportion of fucoxanthin, indicating its potential role in photoprotection. To investigate this, pure fucoxanthin was isolated from 10 seaweed species that grow under prevailing extreme conditions, and its antioxidant, UV-protective, anti-elastase, anti-collagenase and anti-tyrosinase properties were evaluated. Significant interspecies variation was observed in both yield and bioactivity, with seaweeds surviving extreme summer conditions exhibiting higher fucoxanthin content and enhanced UV-protective properties. Fucoxanthin isolated from *Canistrocarpus cervicornis*, *Feldmannia mitchelliae*, *Padina gymnospora*, and *Sargassum angustifolium* showed high antioxidant, anti-elastase, anti-collagenase, anti-tyrosinase and UV- protection properties. Structural characterization by NMR revealed variations in the R group among the fucoxanthin isolates. Fucoxanthin derivatives bearing R group –CH_2_OCO(CH_2_)_4_CH_3_ (hexanoyloxyfucoxanthin) demonstrated superior bioactivity compared to those containing –CH_2_OCO(CH_2_)_2_CH_3_ (butanoyloxyfucoxanthin) and parent compound containing –CH_3_ (fucoxanthin), indicating a structure–activity relationship. Stability studies have shown that fucoxanthin remains stable at temperatures up to 70 °C and is resistant to oxidizing agents such as H_2_O_2_. However, it is susceptible to high-intensity artificial UV-radiation and is highly sensitive to pH changes above 5. Collectively, these findings highlight the adaptive structural diversity of fucoxanthin from seaweeds of the Arabian Gulf and emphasize the importance of species selection in maximizing bioactivity.

## 1. Introduction

Marine macroalgae are rich sources of bioactive compounds with diverse biological activities. Studies have identified sulfated polysaccharides, carotenoids, phenolic compounds, phlorotannins, phytosterols, and bioactive proteins as key constituents of macroalgae that exhibit potential pharmaceutical, nutraceutical, and cosmeceutical applications [1,2]. Among bioactive compounds, the marine carotenoid fucoxanthin has attracted significant attention due to its health-promoting properties [3]. Fucoxanthin constitutes 40–70% of the total carotenoids in brown algae [1,2,4], where it functions as a light-harvesting and energy-transferring pigment within the fucoxanthin–chlorophyll a-protein complex in the thylakoid membranes [5,6]. Numerous studies have demonstrated the bioactive properties of fucoxanthin, including anticancer [7,8], antidiabetic [9], anti-obesity [10], anti-inflammatory [11], antioxidant [12], and hepatoprotective effects [3]. Interest in fucoxanthin as a marine-derived ingredient for cosmetic and cosmeceutical formulations stems from its antioxidant, anti-inflammatory, photoprotective, and anti-aging properties [13]. Studies in skin cell models have shown that fucoxanthin can reduce oxidative stress, promote procollagen synthesis, inhibit matrix metalloproteinases, and suppress melanin production [14,15]. Animal studies have also demonstrated protection against ultraviolet-induced skin damage, inflammation, and wrinkle formation [15,16,17]. Because of these valuable health benefits, the global market value of fucoxanthin is estimated at USD 209.45 million. It is projected to grow at a 5% annual rate over the next five years [18]. This increasing demand highlights the commercial significance of fucoxanthin in the food, pharmaceutical, and cosmetic industries.

Fucoxanthin has been isolated from several species of brown seaweed, and most studies have been conducted in Southeast Asia and Europe [12,19,20]. Several studies have shown that fucoxanthin content varies with extrinsic factors, such as environmental conditions (temperature, light, nutrient levels), and with intrinsic factors, such as species [12,20,21,22,23,24]. Although the beneficial properties of fucoxanthin isolated from other parts of the ocean are well documented, only limited information is available on the content and bioactivity of fucoxanthin from seaweeds in the Arabian Gulf [25]. The Kuwait coast of the Arabian Gulf is a unique marine environment characterized by a pronounced seasonal range in water temperature (13–36 °C), salinity (36–44 ppt), and UV index (8–10) [26,27,28]. Despite these harsh environmental conditions, this region supports 131 marine macroalgal species, including 35 brown algal species [29]. Sea surface temperature plays a crucial role in determining the distribution patterns of these seaweeds, with most species appearing frequently during winter, when water temperatures are lower. In contrast, only a few seaweed species adapted to high water temperatures can thrive in the summer [29]. Macroalgae from this region possess a unique phenolic composition and are richer in flavonoids than their temperate counterparts, which are rich in phlorotannins [27]. Most seaweeds in this region contain hydroquinone, a known skin-whitening agent [27]. This may be an adaptation of these macroalgae to survive the extreme climatic conditions in this region. In our studies on the pigment profile of macroalgae from this region, we observed that macroalgal species that survive the extreme summer months with a high UV index (10) have a high percentage of fucoxanthin [30]. This led to the hypothesis that fucoxanthin may have UV-protective properties. Hence, the present study aimed to isolate pure fucoxanthin from 10 seaweed species collected from the Kuwait coast of the Arabian Gulf and to compare its bioactivity, including antioxidant, UV-protective, anti-collagenase, anti-elastase, and anti-tyrosinase properties.

Few studies have examined the structural variations in fucoxanthin that may arise during post-harvest storage and biological transformation. Known structural variants include cis/trans geometric isomers, which form during light exposure or thermal processing [31,32]. Fucoxanthinol, the major deacetylated metabolite of fucoxanthin generated enzymatically in vivo, and amarouciaxanthin A, a further metabolized product of fucoxanthinol, were shown to have higher bioactive properties [33]. These derivatives have been studied mainly in the context of metabolism or degradation, and comparative analyses of naturally occurring structural variants remain limited. Such variations may influence fucoxanthin’s bioactivity and UV-protective function, yet this aspect has received comparatively little attention. Hence, in the present study, the structural characterization of the isolated fucoxanthin was performed using 1D- and 2D NMR spectroscopy. In addition, stability studies were conducted to assess its suitability as a cosmetic agent.

## 2. Results

### 2.1. Yield and Purity of Fucoxanthin from Different Seaweeds

**Figure 1:**
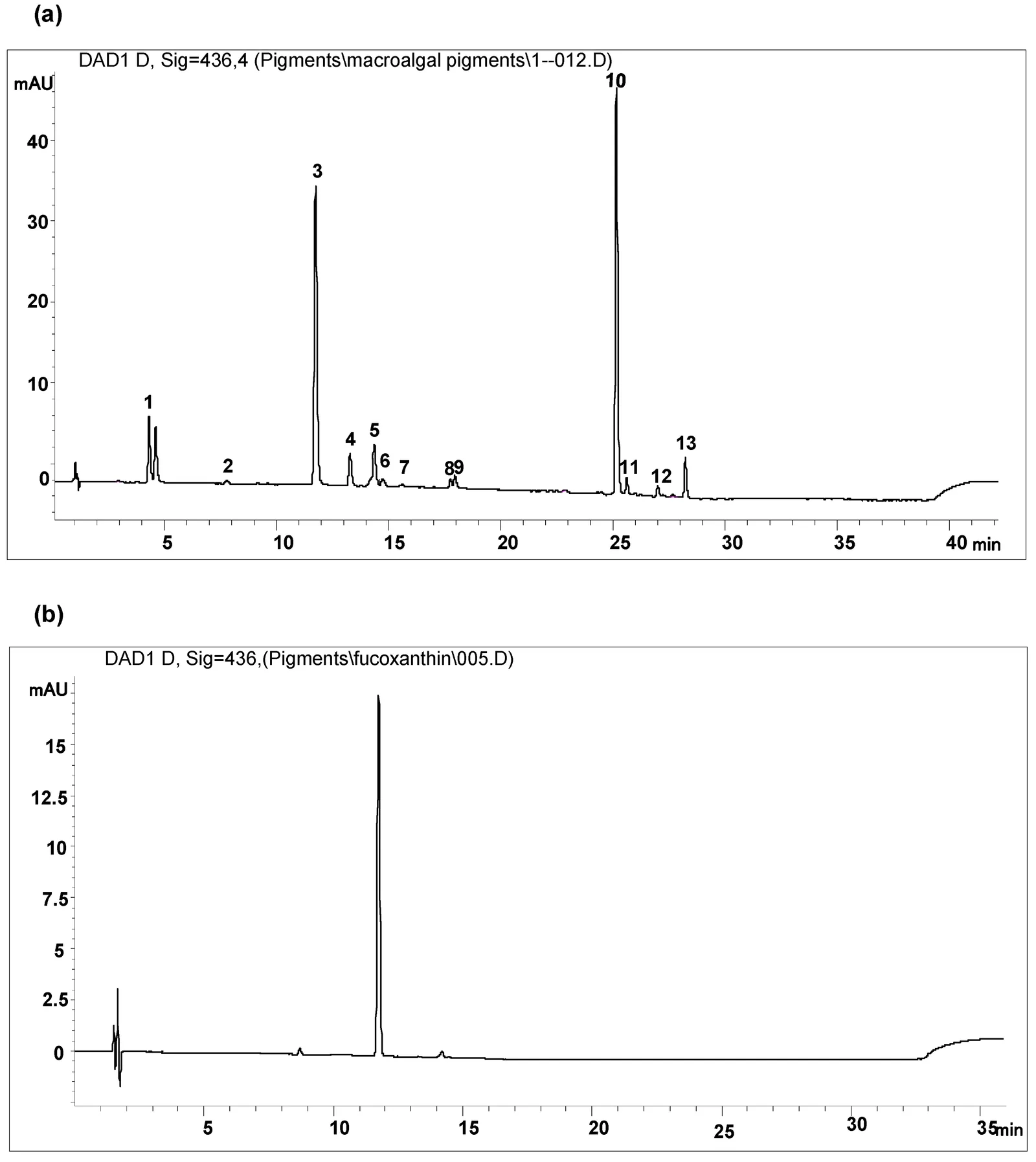
The representative chromatograms of pigment profile (**a**) and purified fucoxanthin (**b**) from *C. sinuosa*. The numbers represent: 1: chlorophyll c2, 2: peridinin, 3: fucoxanthin, 4: astaxanthin, 5: diadinoxanthin, 6: dinoxanthin, 7: alloxanthin, 8: leutin, 9: zeaxanthin, 10: chlorophyll a, 11: divinyl chlorophyll a, 12: α-carotene, 13: β-carotene.

Fucoxanthin isolated by low-temperature crystallization was further purified twice on silica gel columns using hexane: acetone (7:3) as the eluent. HPLC chromatograms of purified fucoxanthin were without co-eluting carotenoids or other pigment classes, confirming the high purity (Figure 1a,b). The chromatogram of purified fucoxanthin showed one dominant peak accounting for 98% of the total integrated peak area, with two minor peaks together accounting for the remaining 2% (Figure 1b), representing an HPLC area purity of 98%. However, isolated fucoxanthin showed slight variation in retention time compared with the fucoxanthin standard (Figure S12). Hence, the isolated fucoxanthins were subjected to ^1^H NMR and 2D NMR (HSQC, HMBC) analysis, and the results are discussed in Section 2.6.

**Figure 2:**
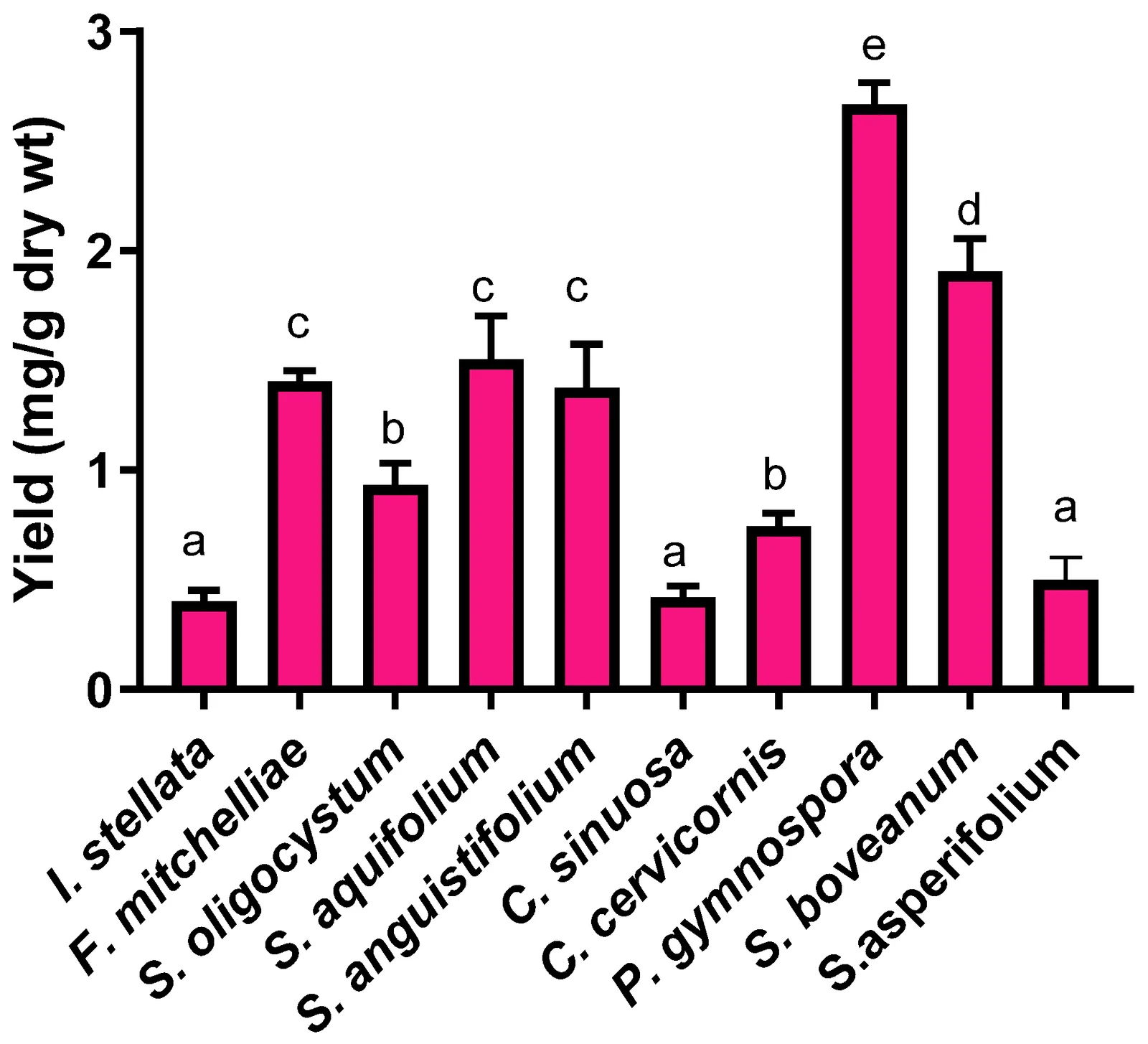
Yield of isolated fucoxanthin from ten species of seaweeds from the Kuwait coast. Values are the mean ± SD of three independent extractions (*n* = 3). The letters a, b, c, d, and e represent significant differences in yield among species (same letters indicate no significant differences).

The yield of the fucoxanthin varied significantly among the 10 species of seaweeds studied, ranging from 0.3 to 2.7 mg/g dry weight (Figure 2). The highest yield was observed in *P. gymnospora* (2.7 ± 0.2), followed by *S. boveanum*, *S. aquifolium*, *F. mitchelliae*, *S. angustifolium*, *S. oligocystum*, and *C. cervicornis. S. asperifolium*, *I. stellata*, and *C. sinuosa* showed low fucoxanthin contents, and there was no significant difference in fucoxanthin content among these species.

### 2.2. Antioxidant Activity of Isolated Fucoxanthin

**Figure 3:**
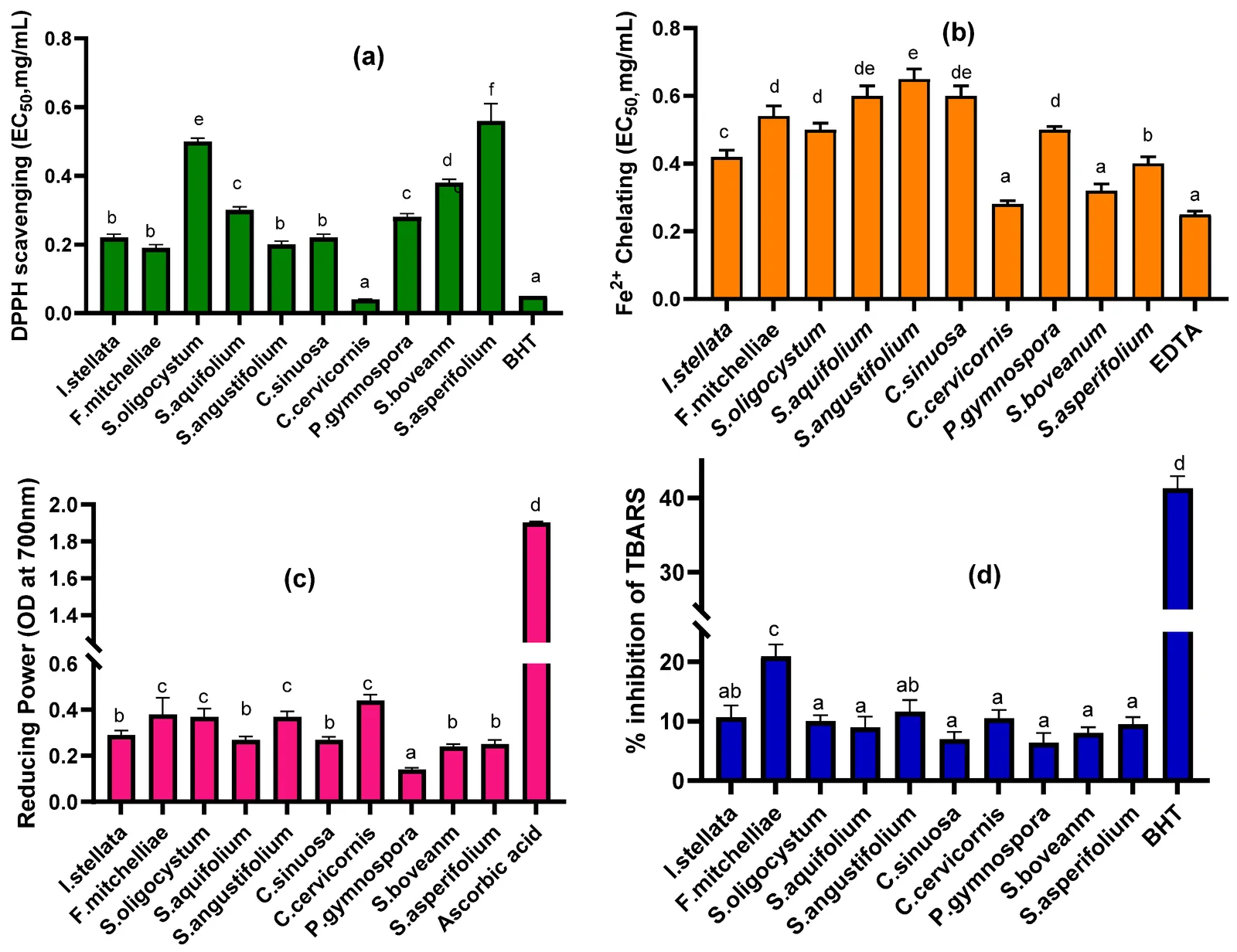
Antioxidant activity of fucoxanthin isolated from the Kuwait coast of the Arabian Gulf: (**a**) DPPH radical-scavenging activity, (**b**) metal chelating activity, (**c**) reducing power, and (**d**) inhibition of oxidation in the liposome model system. Values are the mean ± SD of the triplicate analysis (*n* = 3). The letters a, b, c, d, e and f represent significant differences in activity across the species (same letters indicate no significant differences).

The antioxidant activity of isolated fucoxanthin was evaluated using α-diphenyl-β-picrylhydrazyl (DPPH) radical-scavenging, metal chelation, reducing power, and lipid oxidation inhibition in a liposome model system (Figure 3a–d). The radical-scavenging properties of isolated fucoxanthin showed species-specific variation (Figure 3a). In all seaweeds, the radical-scavenging activity of fucoxanthin was concentration-dependent, and the activity increased with increasing concentration (Table S1). The highest radical-scavenging activity was observed for fucoxanthin from *C. cervicornis* (lowest EC_50_, 0.04 mg/mL) and was better than that of the synthetic antioxidant butylated hydroxytoluene (BHT) (EC_50_, 0.05 mg/mL) (Figure 3a). This was followed by fucoxanthin, isolated from *F. mitchelliae*, *S. angustifolium*, and *I. stellata*. Fucoxantin from *S. asperifolium* showed the lowest radical scavenging activity (highest EC_50_, 0.56 mg/mL), followed by *S. oligocystum*.

The iron-chelating activity also showed a concentration-dependent increase (Table S2). The iron-chelating activity (EC_50_) of the fucoxanthin isolated from different seaweeds varied from 0.28 ± 0.01 to 0.65 ± 0.02 mg/mL (Figure 3b). Fucoxanthin from *C. cervicornis* and *S. boveanum* showed the highest iron-chelating activity, with the lowest EC_50_ value, and there was no significant difference (*p* > 0.05) when compared with the known metal chelator, ethylenediaminetetraacetic acid (EDTA). The iron-chelating activity of fucoxanthin from other species was significantly lower than that of EDTA (*p* < 0.001).

The reducing power of pure fucoxanthin (0.5 mg/mL) is shown in Figure 3c. It varied by species, ranging from 0.14 ± 0.0 to 0.44 ± 0.03. Among the species, fucoxanthin isolated from *C. cervicornis*, *F. mitchelliae*, *S. oligocystum*, and *S. angustifolium* exhibited a relatively higher reducing power than the other species. However, for all other fucoxanthins, the reducing power was less than 0.3.

The inhibition of lipid oxidation by fucoxanthin (0.1 mg/mL) in the liposome model system is shown in Figure 3d. Among the species, the highest inhibition of thiobarbituric acid-reactive substances (TBARS) formation was observed with fucoxanthin isolated from *F. mitchelliae*. Fucoxanthin obtained from *C. sinuosa* and *P. gymnospora* exhibited the lowest inhibition of TBARS formation. There were no significant (*p* > 0.05) differences in activity compared to fucoxanthin from other species. BHT at a concentration of 0.1 mg/mL showed an inhibition of 41.3 ± 1.6%, which was significantly (*p* < 0.001) higher than that of fucoxanthin.

### 2.3. UV-Protective Effect of Isolated Fucoxanthin on E. coli Model System

**Figure 4:**
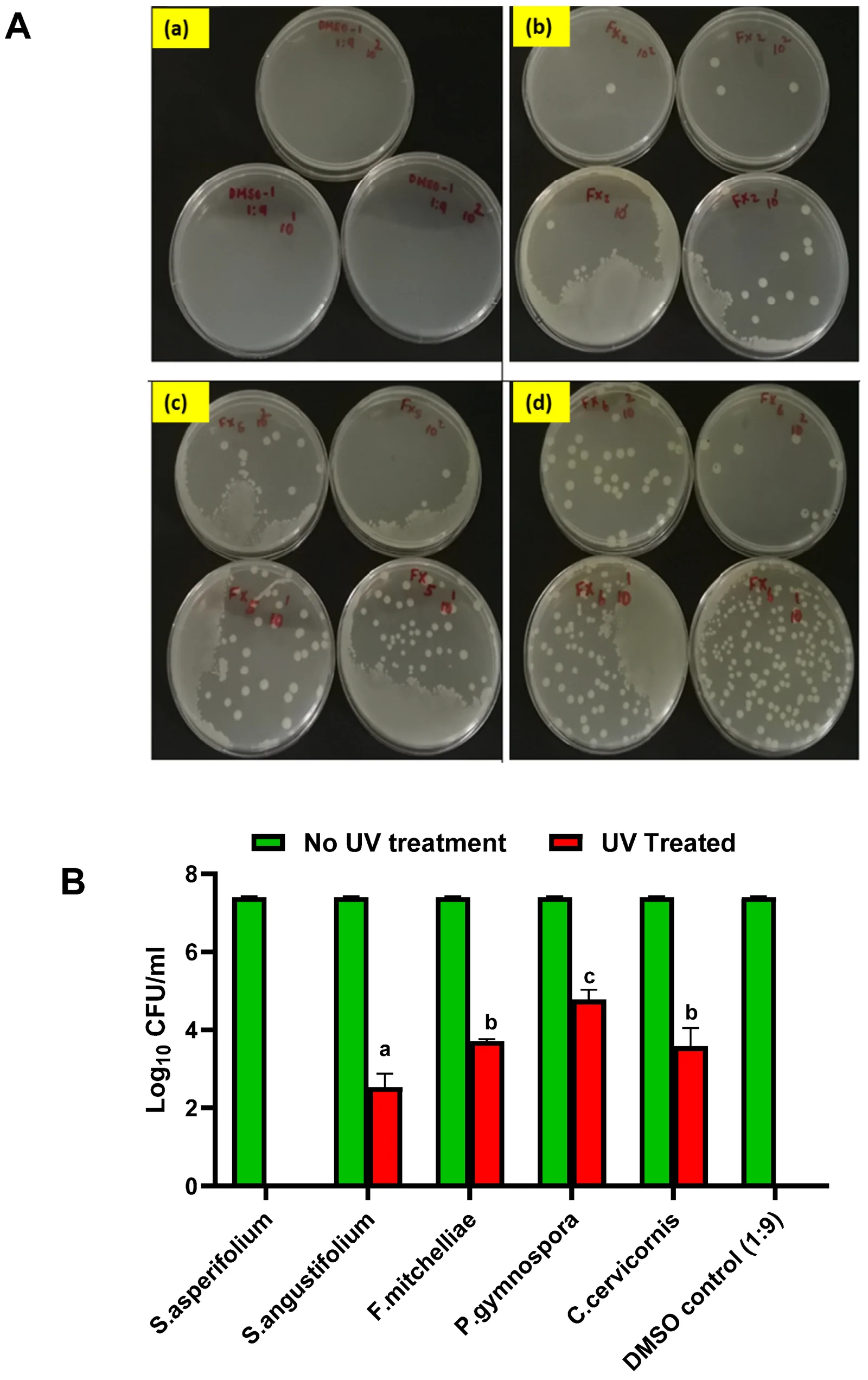
(**A**) Representative images showing the UV-protective effects of fucoxanthin in the *E. coli* model system: (**a**) DMSO control, (**b**) fucoxanthin from *S. angustifolium*, (**c**) fucoxanthin from *P. gymnospora*, and (**d**) fucoxanthin from *C. cervicornis.* (**B**) Protective effect of isolated pure fucoxanthin on *E. coli* against UVradiation. Values are the mean ± SD of the triplicate analysis (*n* = 3). The letters a, b, and c represent significant differences in UV-protective activity across the species (same letters indicate no significant differences).

The UV-protective effect of fucoxanthin isolated from five species of seaweeds, namely, *C. cervicornis*, *F. mitchelliae*, *S. angustifolium*, *P. gymnospora*, and *S. asperifolium*, is shown in Figure 4. The control with dimethyl sulfoxide (DMSO) (without fucoxanthin) did not protect *E. coli* against UV radiation (Figure 4(Aa)). In contrast, fucoxanthin protects *E. coli* against high UV radiation (Figure 4(Ab–Ad)). In the absence of UV, DMSO caused a one-log reduction in *E. coli* survival, suggesting a mild bactericidal effect. Among the five species tested, fucoxanthin from *P. gymnospora* showed the highest protection against UV radiation (Figure 4(Ac),B). This was followed by fucoxanthin from *C. cervicornis*, *F. mitchelliae*, and *S. angustifolium*. However, fucoxanthin from *S. asperifolium* did not protect *E. coli* from UV radiation (Figure 4B). These results indicated that the protective effects of fucoxanthin against UV radiation varied across species, with some exhibiting greater protection than others (Figure 4B).

### 2.4. Anti-Collagenase and Anti-Elastase Activity of Isolated Fucoxanthin

**Figure 5:**
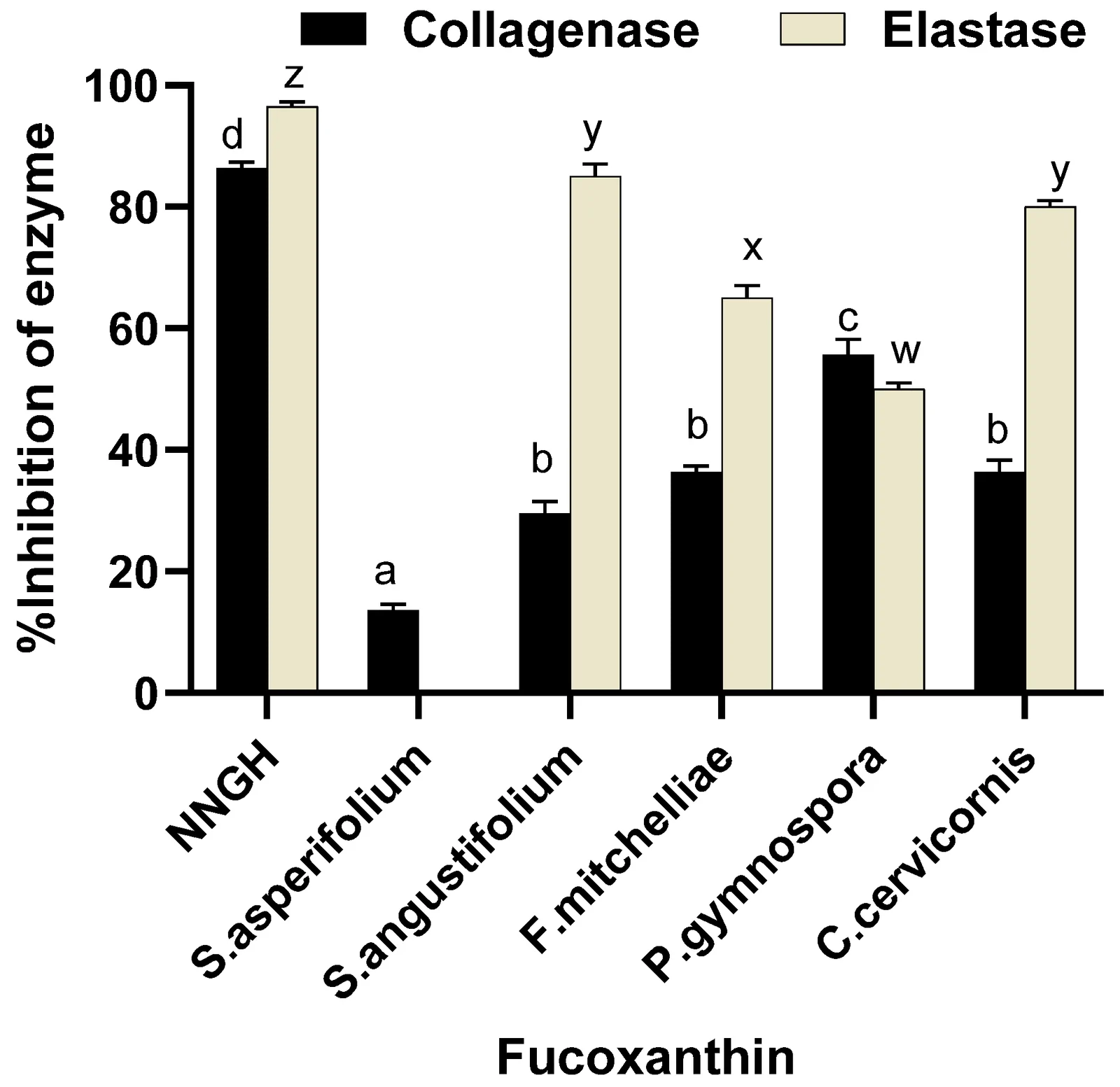
The collagenase and elastase inhibition properties of isolated fucoxanthin. Values are the mean ± SD of triplicate analyses (*n* = 3). The letters a, b, c, and d represent significant differences in collagenase inhibition across the species, and the letters w, x, y, and z represent significant differences in elastase inhibition across the species (same letters indicate no significant differences).

The anti-wrinkle properties of fucoxanthin were evaluated using collagenase and elastase inhibition assays (Figure 5). Fucoxanthin isolated from *S. angustifolium* exhibited the highest elastase inhibition (85%), which closely approached the performance of the known matrix metalloproteinase (MMP) inhibitor N-Isobutyl-N-(4-methoxyphenylsulfonyl)glycyl hydroxamic acid (NNGH) (96% elastase inhibition). On the other hand, fucoxanthin from *C. cervicornis* and *F. mitchelliae* exhibited elastase-inhibitory activities of 80% and 65%, respectively. *P. gymnospora* showed moderate elastase inhibition (50%), whereas *S. asperifolium* showed no elastase inhibition. For collagenase inhibition, *P. gymnospora* showed the highest activity (56%), outperforming fucoxanthin from other species (Figure 5). *F. mitchelliae* and *C. cervicornis* exhibited 40% collagenase inhibition, whereas the known MMP inhibitor, NNGH, showed a remarkable (86%) collagenase inhibition. *S. angustifolium* and *S. asperifolium* displayed lower collagenase inhibition activities of 30% and 13%, respectively (Figure 5).

### 2.5. Anti-Tyrosinase Activity of Isolated Fucoxanthin

**Figure 6:**
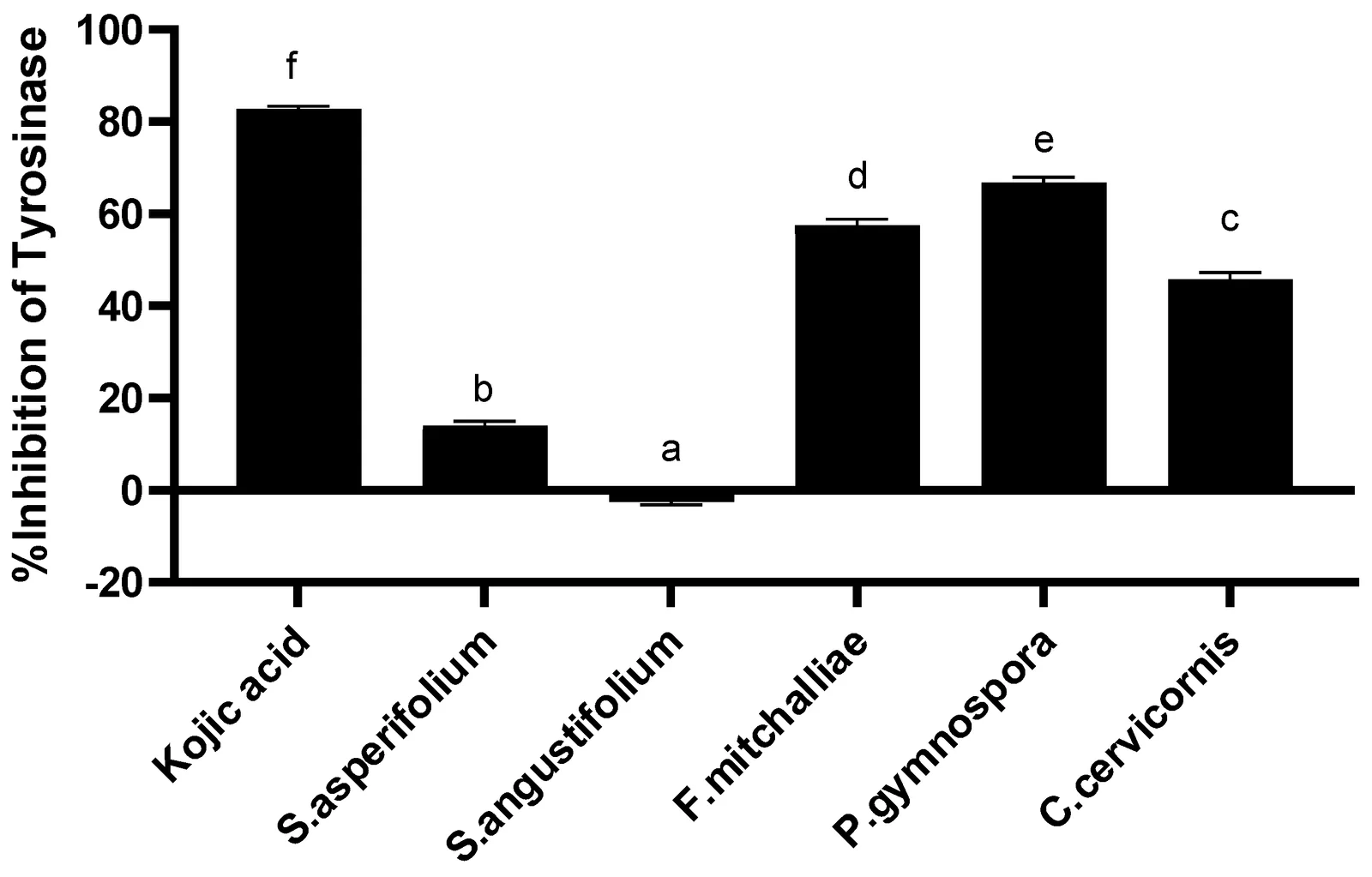
The anti−tyrosinase activity of fucoxanthin isolated from the Kuwait seaweeds. Values are the mean ± SD of triplicate analyses (*n* = 3). The letters a, b, c, d, e, and f denote significant differences in tyrosinase inhibition among species (same letters indicate no significant differences).

The anti-tyrosinase activity of fucoxanthin is shown in Figure 6. Kojic acid, a well-known tyrosinase inhibitor [34], was used as a positive control and showed an inhibition rate of 83% (Figure 6). Among the fucoxanthin samples, *P. gymnospora* exhibited the highest tyrosinase inhibition (67%), followed by *F. mitchelliae* (57%) and *C. cervicornis* (50%). Low tyrosinase inhibition was observed in *S. asperifolium* (14%), whereas fucoxanthin from *S. angustifolium* showed no tyrosinase inhibition; instead, it increased tyrosinase activity.

### 2.6. Structural Studies on the Isolated Fucoxanthin

**Figure 7:**
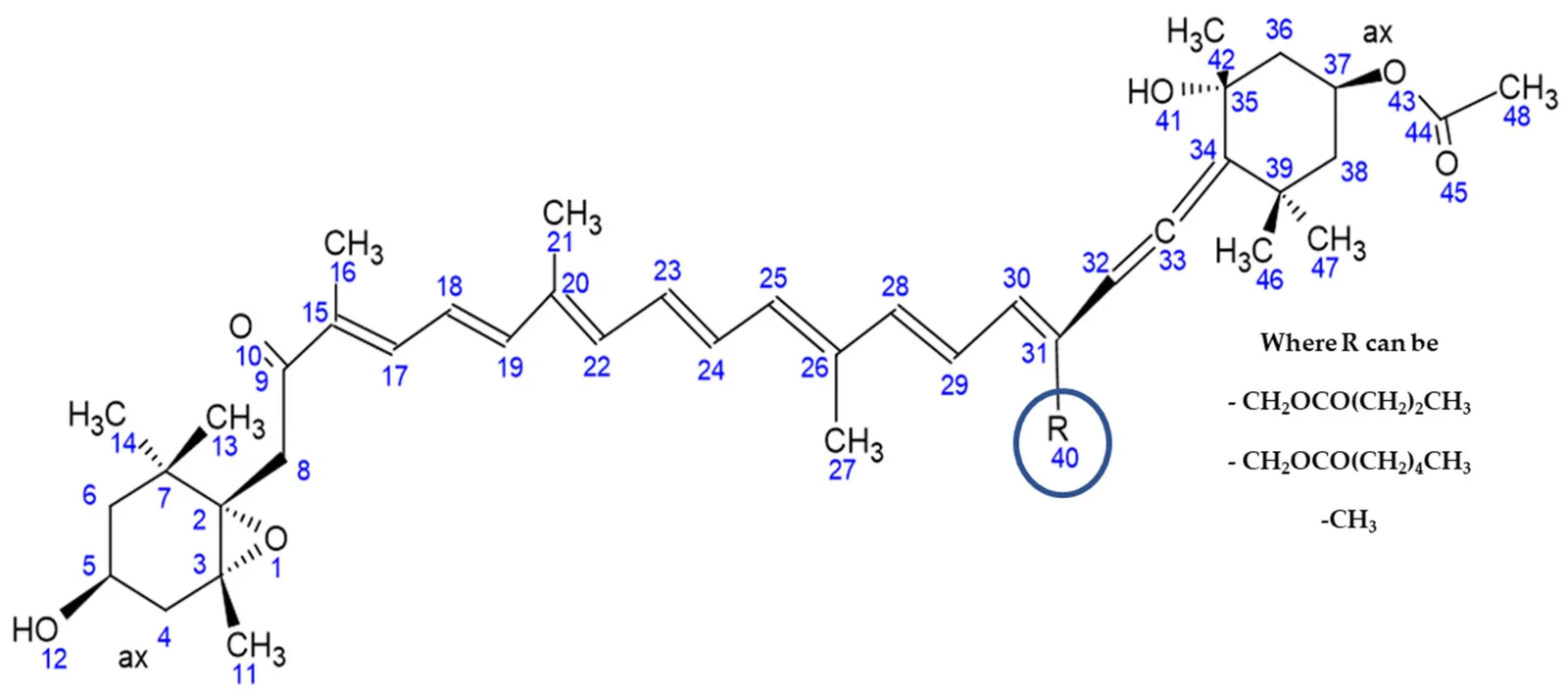
The structure of fucoxanthin proposed from the NMR analysis of the present study with atom numbers.

NMR analysis of fucoxanthin obtained from nine brown seaweed species was performed, as shown in Figures S1–S11 and Table 1. Assignments were made according to the method of Englert et al. [35]. Though the backbone structure of the fucoxanthin molecule is preserved, the fucoxanthin molecules from seaweeds of the Kuwait coast differ in the R group attached to it (Figure 7, Table 1). Based on the difference in the R group (Figure 7, Table 1), seaweed species can be categorized into three groups: I) fucoxanthin from *C. sinuosa*, *I. stellata*, *S. angustifolium*, and *P. gymnospora* forms the first group, II) fucoxanthin from *S. aquifolium, S. boveanum*, *F. mitchelliae* and *C. cervicornis* constitutes the second group, and III) fucoxanthin from *S. oligocystum* is distinct from the groups mentioned above. The first fucoxanthin group has R as CH_2_OCO(CH_2_)_2_CH_3_ (butanoyloxymethyl), whereas the second group has R as CH_2_OCO(CH_2_)_4_CH_3_ (hexanoyloxymethyl). The R group of fucoxanthin from *S. oligocystum* is a –CH3 group (methyl) (Figure 7, Table 1). The canonical structure of fucoxanthin (C_42_H_58_O_6_), containing a methyl group at the R group, is well established, and it corresponds to the fucoxanthin isolated from *S. oligocystum* in the present study, where the substituent (R) is –CH_3_. The other fucoxanthin groups with different R groups, as mentioned above, can therefore be more accurately described as fucoxanthin derivatives or structural variants, rather than being identical to the canonical fucoxanthin molecule. The slight variations in HPLC retention times observed for some isolates from the Sigma fucoxanthin (Figure S12) might be due to these differences in R group substitutions in the fucoxanthin molecules, which influence polarity and chromatographic behavior.

Fucoxanthin samples from *C. sinuosa*, *I. stellata*, *S. angustifolium*, and *S. aquifolium* were in the *cis* form (indicated by the peak at 4.58 ppm). The fucoxanthin sample from *S. oligocystum* lacked resonances in the 9.40–9.65 ppm region. Therefore, it does not have a -CHO group, which is the most remarkable difference in the chemical structure of this fucoxanthin sample compared to fucoxanthin molecules from other species.

### 2.7. Stability of Isolated Fucoxanthin

**Figure 8:**
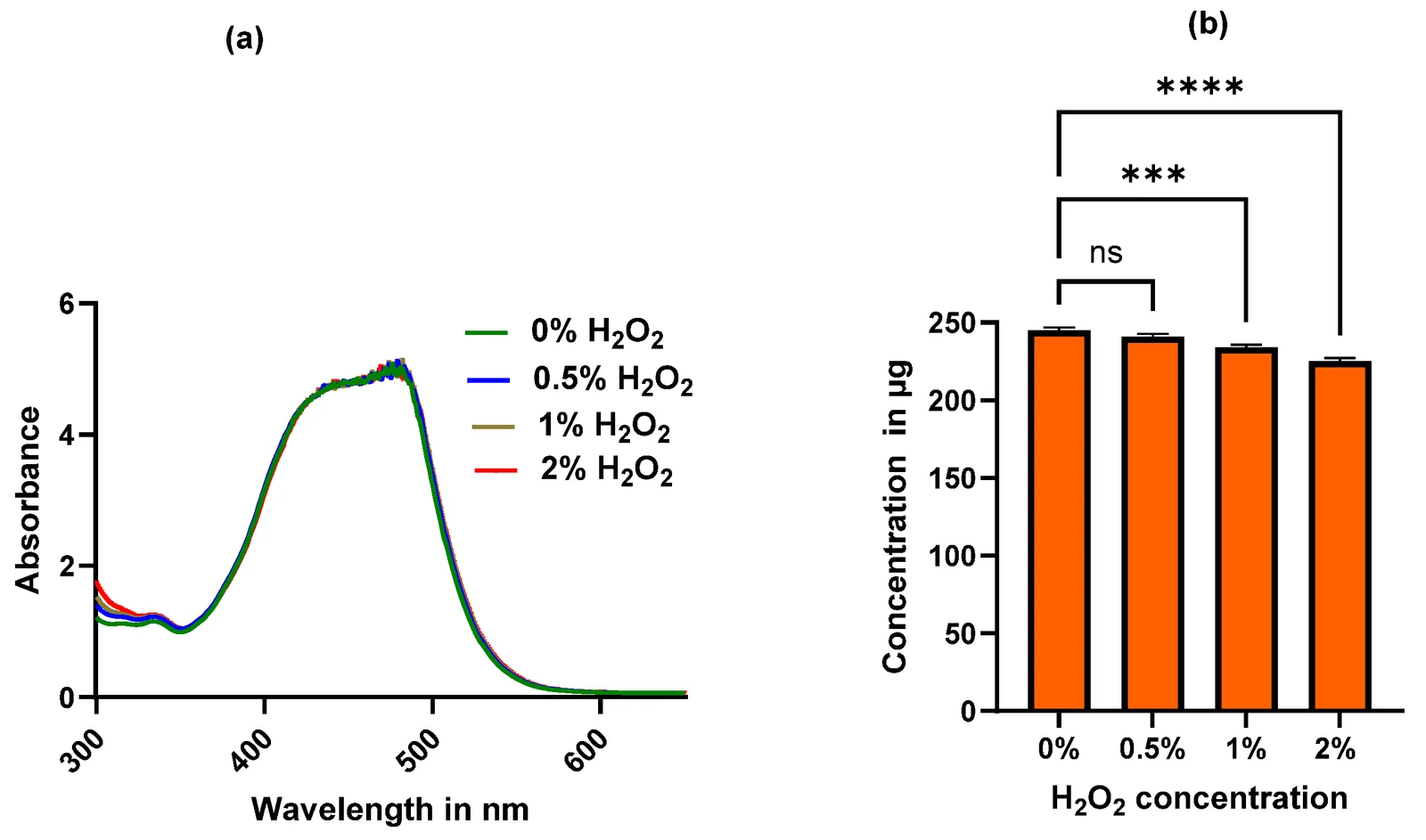
H_2_O_2_ stability of the isolated fucoxanthin (**a**) UV-Vis spectra; (**b**) HPLC quantification of fucoxanthin. Values are the mean ± SD of triplicate analyses (*n* = 3). The symbols represent significant differences: *** *p* = 0.0004 and **** *p* < 0.0001.

For the stability studies, fucoxanthin isolated from *S. angustifolium* was used. The stability of fucoxanthin under oxidative conditions was assessed by exposing it to varying concentrations of hydrogen peroxide (H_2_O_2_) (0.5%, 1%, and 2%) (Figure 8a,b). UV-visible spectral analysis showed no significant changes upon the addition of H_2_O_2_ (Figure 8a). However, HPLC analysis provides more detailed insight into its stability. At 0.5% H_2_O_2_, fucoxanthin levels remained unaffected. In contrast, higher concentrations of H_2_O_2_ (1% and 2%) showed reductions of 4.5% and 8.1%, respectively, compared to the control sample (Figure 8b).

**Figure 9:**
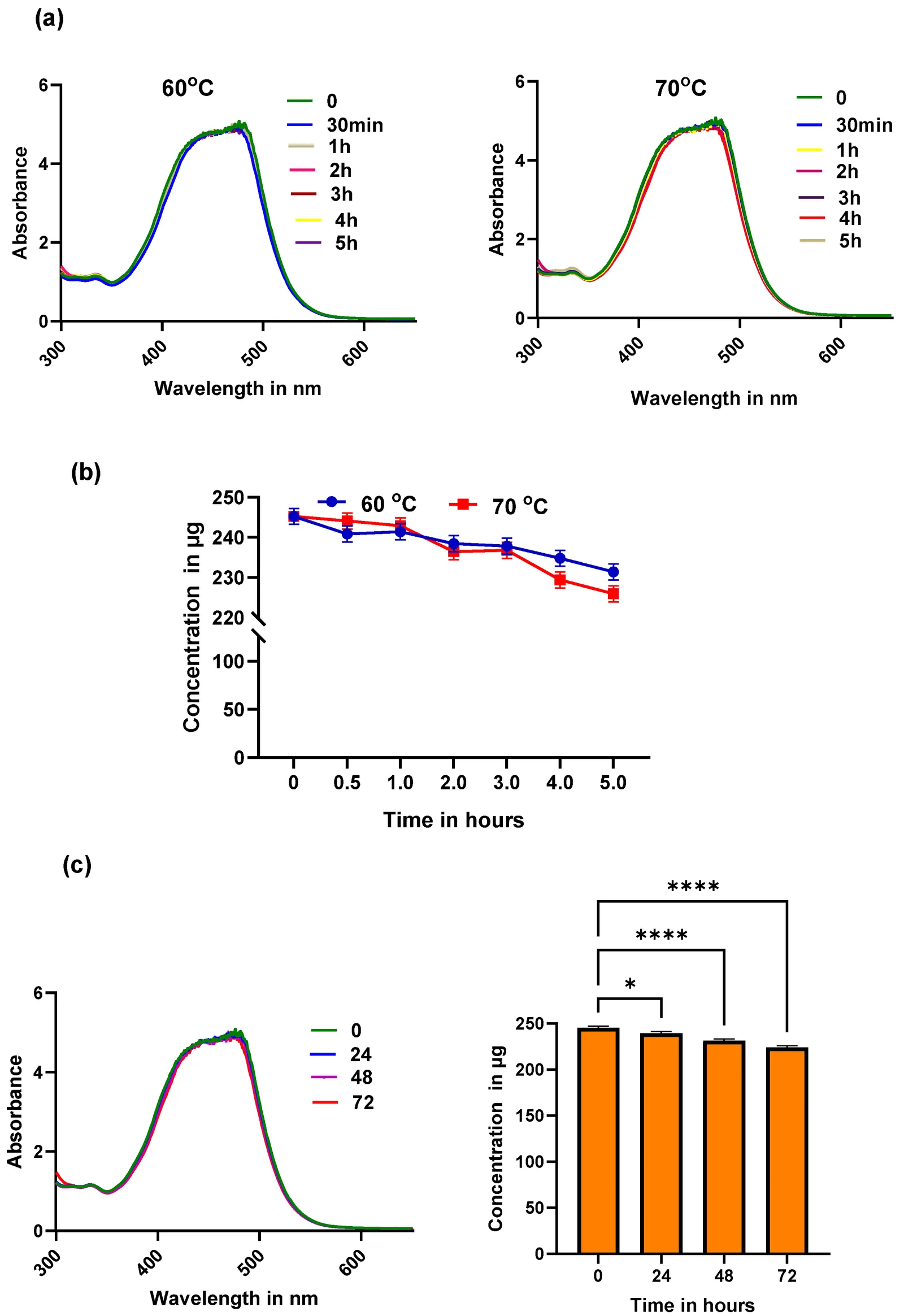
Thermal stability of isolated fucoxanthin showing (**a**) the UV-Vis spectra of fucoxanthin heated to 60 and 70 °C, (**b**) the HPLC quantification of fucoxanthin at 60 and 70 °C, and (**c**) the UV-Vis spectra and the HPLC quantification of fucoxanthin at room temperature. Values are the mean ± SD of triplicate analyses (*n* = 3). * *p* = 0.0166; **** *p* < 0.0001.

The thermal stability of fucoxanthin was evaluated at 60 °C, 70 °C, and room temperature (20 °C) (Figure 9a–c). The results showed that heating at 60 °C and 70 °C led to 5% and 7% reductions in fucoxanthin content, respectively, over 5 h. At 60 °C, no significant changes were observed during the first hour. However, from the second hour onward, a gradual decline in fucoxanthin levels was observed, with 4–5% reductions at 60 °C and 6–7% at 70 °C over 5 h, respectively (Figure 9b). Storage at room temperature (20 °C) resulted in only an 8% reduction in fucoxanthin content within three days compared to the control (Figure 9c). Despite these losses, there were no remarkable changes in the UV-Vis spectra at 60 °C, 70 °C, or 20 °C (Figure 9a,c).

**Figure 10:**
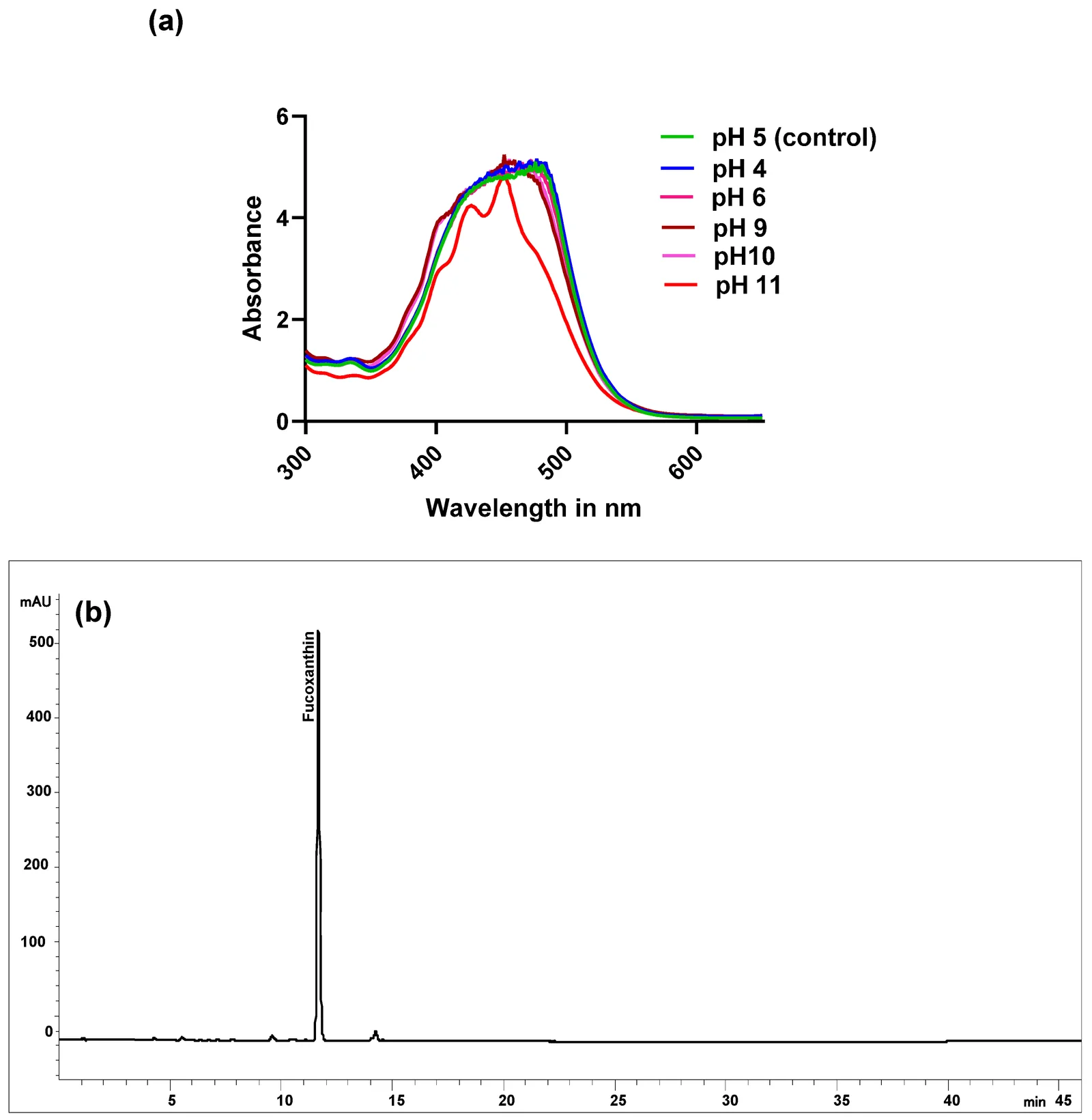

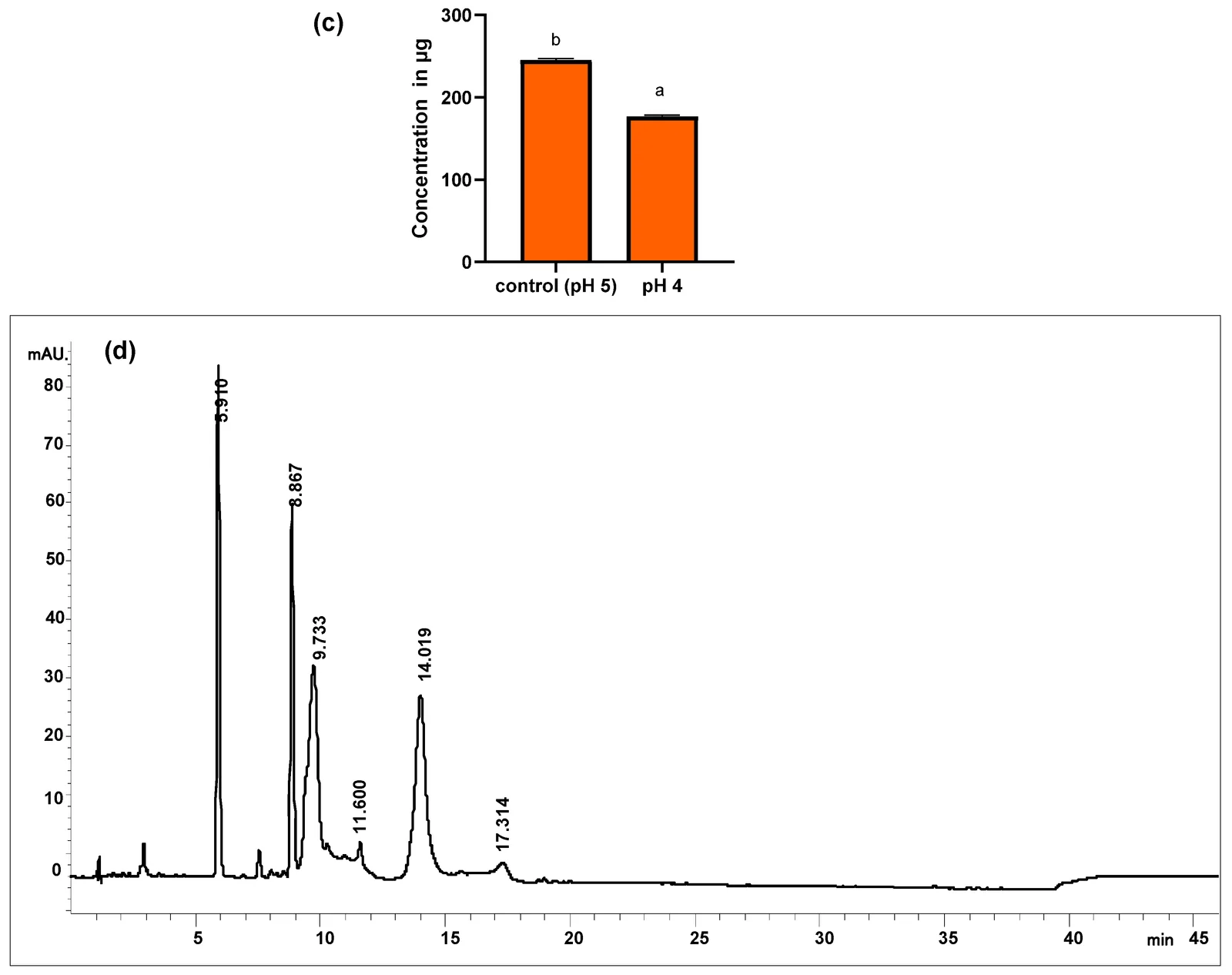
pH stability of fucoxanthin. (**a**) UV-Vis spectra of fucoxanthin at different pH values; (**b**) HPLC chromatogram of pure fucoxanthin from *S. angustifolium* at pH 5 (control); (**c**) HPLC quantification of fucoxanthin; (**d**) representative HPLC chromatogram showing fucoxanthin degradation at pH 6. Values are the mean ± SD of triplicate analyses (*n* = 3). The letters a and b represent significant differences in pH exposure (same letters indicate no significant differences).

The stability of fucoxanthin over a pH range from 4 to 11 using pure fucoxanthin was investigated (Figure 10a–d). The initial pH of the fucoxanthin solution was five (control). When the pH was lowered to four, no significant changes were observed in the UV-Vis spectrum (Figure 10a). However, HPLC analysis revealed a considerable (27%) reduction in fucoxanthin content compared to the control (Figure 10b). Conversely, raising the pH to 6 and above resulted in noticeable changes in the UV-Vis spectra and HPLC chromatograms (Figure 10a,c,d). At pH ≥6, quantification via HPLC became challenging as fucoxanthin degraded into multiple peaks (Figure 10c,d).

**Figure 11:**
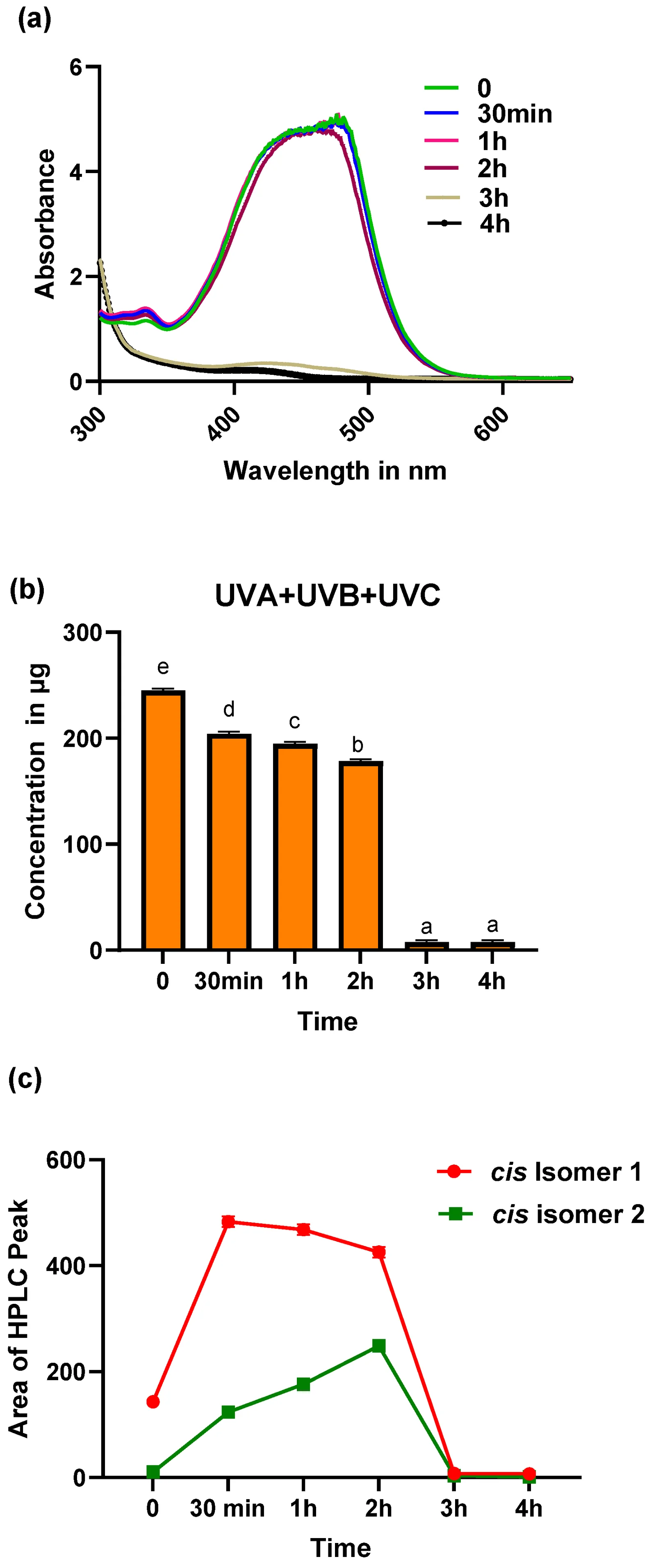
The UV stability of fucoxanthin is shown in (**a**) the UV-Vis spectra of fucoxanthin treated with high UV at different times, (**b**) the HPLC quantification of fucoxanthin, and (**c**) the HPLC quantification of isomers of fucoxanthin. Values are the mean ± SD of triplicate analyses (*n* = 3). The letters a, b, c, d, and e represent significant differences (same letters indicate no significant differences).

The stability of fucoxanthin under high-intensity UV radiation was investigated (Figure 11a,b). Initially, the UV-Vis spectra showed no significant changes within the first hour of exposure (Figure 11a). However, noticeable spectral alterations were observed after two hours of exposure (Figure 11b). After the third and fourth hours of exposure, fucoxanthin was completely degraded, demonstrating rapid breakdown under prolonged high-intensity UV exposure (Figure 11a,b). HPLC quantification supported these findings, showing a gradual reduction in fucoxanthin content (16% after 30 min, followed by 20% and 27% reductions over the next 2 h of UV exposure). Interestingly, by the third hour, a 97% reduction in fucoxanthin levels was observed, which persisted even after four hours of exposure (Figure 11b). Interestingly, two minor peaks tentatively assigned to cis-fucoxanthin isomers, designated cis1 and cis2, appeared in the HPLC chromatogram alongside a decrease in fucoxanthin content (Figure 11c).

## 3. Discussion

### 3.1. Yield of Fucoxanthin from Different Seaweeds

The yield of pure fucoxanthin isolated in the present study varied significantly among the 10 seaweed species tested and was far higher than that reported for other seaweeds from the Iranian coast and the Indian Ocean [25,36]. Similar intra- and interspecies variation in fucoxanthin yield has been reported in several studies [1,4,12]. The yield of 85–90% fucoxanthin reported from *L. digitata*, *F. vesiculosus*, and *F. serratus* from the Danish coast was 5.1, 5.0, and 1.13 mg/g, respectively [12]. In the present study, *P. gymnospora* had the highest fucoxanthin yield and was the most abundant species along the Kuwait coast during the summer. This may be an adaptation to harsh environmental conditions, including a high UV index. Fucoxanthin has been reported to possess UV-B protective properties [16,37,38]. Variations in fucoxanthin yield among different seaweeds may be due to the combined effects of species, geographic location, and season [4,39,40]. These results highlight the importance of selecting the appropriate species and season for fucoxanthin extraction to maximize the yield of this valuable compound.

### 3.2. Antioxidant Activity of Isolated Fucoxanthin

The antioxidant activity of isolated fucoxanthin varied among the species. The radical-scavenging activity of fucoxanthin was concentration-dependent. Similar concentration-dependent trends in the radical-scavenging activity of fucoxanthin and its isomers have been reported previously [12,41,42], owing to its enhanced hydrogen-donating ability at higher concentrations. Fucoxanthin and fucoxanthinol have been reported to have higher DPPH radical-scavenging activities than halocynthiaxanthin and other carotenoids [43,44]. The excellent antioxidant activity of fucoxanthin is believed to be due to the molecule’s conjugated bonds, which facilitate the transfer of excited electrons to radicals [45]. In addition to the conjugated double bonds, allenic bonds and hydroxyl groups in fucoxanthin play a role in neutralizing radicals [43,45]. In the present study, fucoxanthin from *C. cervicornis* showed comparatively higher radical-scavenging and iron-chelating activities than synthetic antioxidants. Similar to our findings, Habeebullah et al. [12] reported an iron-chelating activity comparable to EDTA for 85–90% pure fucoxanthin obtained from Danish seaweeds. However, unlike the present study, fucoxanthin isolated from three different species was combined to study the activity. The results of the present study showed that the iron-chelating activity of fucoxanthin is species-specific. Similar to previous studies [12,41], the reducing power of fucoxanthin from different seaweeds is lower than that of ascorbic acid. In the present study, the inhibition of lipid oxidation by fucoxanthin in the liposome model system was lower than that by BHT. Among fucoxanthins from different species, the highest inhibition of oxidation in the liposome model system was observed for fucoxanthin isolated from *F. mitchelliae.* Although fucoxanthin has been reported to possess in vitro antioxidant properties, studies on its effectiveness in lipid-based systems are limited and often yield conflicting results. For instance, Habeebullah et al. [12] reported the antioxidant activity of fucoxanthin in a 5% fish oil-in-water emulsion, where lipid oxidation was induced by iron. In contrast, Tutour et al. [46] reported a weak antioxidant effect for fucoxanthin in the presence o3.3f AIBN and heptanol at 60 °C, which was less potent than that of vitamin E at equivalent concentrations. Further studies are required to elucidate the behavior of fucoxanthin in lipid-containing systems.

### 3.3. UV-Protective Effect of Isolated Fucoxanthin on E. coli Model System

The *E. coli* model is widely used in photobiology to assess DNA damage, oxidative stress responses, and cell survival, especially when evaluating compounds with potential UV-absorbing or antioxidant properties [47,48]. Although the *E. coli* model was not designed to directly mimic mammalian skin photobiology, these results provide a preliminary, mechanistic screen of the photoprotective capacity of the isolated compounds under highly controlled, reproducible conditions. Fucoxanthin isolated from five seaweed species was evaluated for UV-protection. The selection of these five seaweed species was based on their activity in the antioxidant assay and on their occurrence in nature at the highest UV index. Fucoxanthin from three seaweed species, *C. cervicornis*, *F. mitchelliae*, and *S. angustifolium*, was selected based on its high activity in various antioxidant assays. Fucoxanthin from *P. gymnospora* was selected, as this species was available in peak summer when the UV index was at its maximum (UV index = 10). Fucoxanthin from *S. asperifolium* was selected for comparison because of its lowest antioxidant activity, particularly its radical-scavenging activity.

The high fucoxanthin content and UV-protective properties of fucoxanthin from *P. gymnospora* might be the reason for the survival of *P. gymnospora* during peak summer. The observed UV-protective effects of fucoxanthin from *C. cervicornis*, *F. mitchelliae*, and *S. angustifolium* suggest that these species are potential sources of fucoxanthin for UV protection. The level of protection provided by these species was slightly lower than that of *P. gymnospora* but remained significant compared to the control and other species tested. The lack of UV protection by fucoxanthin from *S. asperifolium* underscores the importance of antioxidant activity, particularly radical-scavenging activity, for UV protection. Fucoxanthin offers UV protection by scavenging intracellular ROS generated by UV radiation [16,39].

### 3.4. Anti-Collagenase and Anti-Elastase Activity of Isolated Fucoxanthin

Elastase and collagenase are the two key enzymes that degrade essential structural proteins in the extracellular matrix. Elastase breaks down elastin, a critical component of connective tissues, whereas collagenase targets collagen, a primary structural protein [49]. These enzymes play important roles in various physiological processes, and their deregulation has been linked to several pathological conditions, including inflammatory diseases, tissue damage, and skin aging [50]. In this study, the inhibition of collagenase and elastase by fucoxanthin varied among species. Fucoxanthin isolated from *S. angustifolium* exhibited the highest elastase inhibition (85%), closely approaching the performance of the known MMP inhibitor NNGH [51]. This highlights the potential of fucoxanthin from *S. angustifolium* as a candidate for further exploration in anti-inflammatory therapy. Fucoxanthin from *P. gymnospora* exhibited the highest collagenase inhibition (56%), outperforming fucoxanthin from other species. However, this was lower than that of NNGH, which showed remarkable (86%) collagenase inhibition. Fucoxanthin has been reported to inhibit members of the MMP family, including MMP-2 and MMP-9, which are involved in cancer biology [52]. Fucoxanthinol, a metabolite of fucoxanthin, inhibits MMP1 (collagenase), which plays an essential role in osteosarcoma cell invasion [53]. Similarly, Urikura et al. [16] found that fucoxanthin effectively inhibited UVB-induced epidermal hypertrophy, a key factor in wrinkle formation, and reduced the expression of vascular endothelial growth factor and matrix metalloproteinase-13. Further studies have shown that topical application of fucoxanthin effectively mitigates sunlight-induced skin aging and reduces wrinkles in UVB-exposed mice [14,15,16,17,37,54]. Direct comparison of our findings with those of other studies is challenging, as most research has been conducted in animal models, with limited studies on the in vitro collagenase- and elastase-inhibitory properties of fucoxanthin. The observed variations in the inhibition of MMP-1 and MMP-12 by fucoxanthin extracted from different seaweed species in our study highlight the importance of selecting an appropriate seaweed source to optimize its therapeutic potential.

### 3.5. Tyrosinase Inhibition Activity of Isolated Fucoxanthin

Tyrosinase is an enzyme involved in the synthesis of melanin pigments that are responsible for human skin, hair, and eye color. Excessive melanin production leads to various skin issues, including hyperpigmentation and melasma [55]. The skin-lightening potential of fucoxanthin was evaluated based on its ability to inhibit tyrosinase activity. Shimoda et al. [56] demonstrated that fucoxanthin reduces tyrosinase activity and melanogenesis in melanoma cells and alleviates UVB-induced pigmentation. Furthermore, it significantly downregulated the expression of COX-2, endothelin A receptors, p75 neurotrophins, melanocortin-1, and tyrosinase-related proteins [56]. The findings of the present study indicated that fucoxanthin from *P. gymnospora* achieved nearly two-thirds of the tyrosinase inhibition observed with kojic acid, underscoring its notable potential to suppress tyrosinase activity. While this study provides valuable insights into the tyrosinase inhibition properties of fucoxanthin, further research is needed to elucidate the underlying mechanisms.

### 3.6. Structure and Activity Relationship of Isolated Fucoxanthin

The NMR analysis of the isolated fucoxanthin revealed the presence of fucoxanthin and structural variants such as butanoyloxyfucoxanthin and hexanoyloxyfucoxanthin in the seaweed species studied. Hexanoyloxyfucoxanthin and butanoyloxyfucoxanthin have been reported in Haptophycean algae such as *Phaeocystis* [57,58]. The presence of these fucoxanthin derivatives from seaweeds of this region might be an adaptation to the extreme conditions prevailing in the Arabian Gulf and warrants further investigation.

Fucoxanthin variants with R of CH_2_OCO(CH_2_)_4_CH_3_ (hexanoyloxyfucoxanthin) exhibited the highest activity in radical scavenging, iron chelation, reducing power, and elastase inhibition. In contrast, variants with R = CH_2_OCO(CH_2_)_2_CH_3_ (butanoyloxyfucoxanthin) displayed notable activity in UV–protection, collagenase inhibition, and anti-tyrosinase assays. Fucoxanthin with R = –CH_3_ (canonical form) showed minimal activity under the tested conditions. Species-specific trends were also observed. For instance, fucoxanthin variants isolated from *C. cervicornis* (R = CH_2_OCO(CH_2_)_4_CH_3_) showed the strongest antioxidant activity and elastase inhibition. At the same time, fucoxanthin variants from *F. mitchelliae* showed superior protection against lipid oxidation in the liposome model system. Fucoxanthin variants isolated from *P. gymnospora* (R of CH_2_OCO(CH_2_)_2_CH_3_) showed the highest UV protection, collagenase inhibition, and anti-tyrosinase activity. Additionally, *trans* isomers of fucoxanthin were more bioactive than the *cis* forms. This aligns with earlier reports that, in general, *trans* versions of fucoxanthin have higher bioactivity, although the *cis* forms are active in some anticancer and anti-inflammatory studies [36,59].

The structure–activity relationships of these fucoxanthin variants can be attributed to physicochemical differences introduced by their R groups. Longer R chains, such as CH_2_OCO(CH_2_)_4_CH_3_, increase hydrophobicity, allowing deeper insertion into lipid bilayers and stronger interactions with hydrophobic enzyme pockets. Higher hydrophobicity also promotes more effective embedding within the lipid bilayer, where lipid oxidation occurs, thereby enhancing antioxidant activity [60]. Elastase possesses a more hydrophobic active site than collagenase and tyrosinase [61], and increased molecular hydrophobicity has been shown to improve elastase-inhibitory potency because of the predominantly hydrophobic nature of its active site and surrounding binding pockets [62]. This likely accounts for the higher antioxidant and elastase-inhibitory activities observed in fucoxanthin variants bearing CH_2_OCO(CH_2_)_4_CH_3_ groups. Conversely, shorter R groups such as CH_2_OCO(CH_2_)_2_CH_3_, reduce steric bulk and slightly increase polarity, which may enhance the hydrogen bonding and binding to the less hydrophobic binding site of collagenase and tyrosinase, as seen with the fucoxanthin-containing R group of CH_2_OCO(CH_2_)_2_CH_3_. These structural variations may also reflect seaweeds’ adaptive responses to the extreme environmental conditions along the Kuwait coast, thereby influencing the biosynthesis of specific forms of fucoxanthin. Further work is needed to clarify how these structural differences relate to specific bioactive mechanisms and ecological adaptation.

### 3.7. Stability of Fucoxanthin

As fucoxanthin has UV-protection properties, assessing its stability under various conditions is essential to determine its suitability for cosmetic or skincare applications. The present study examined the vital parameters of skincare cream preparations and assessed their thermal, pH, UV, and H_2_O_2_ stability.

The results show that 0.5% H_2_O_2_ has no impact on fucoxanthin stability, and at higher concentrations of 1–2% H_2_O_2_ has a minimal impact. Previous studies have shown that fucoxanthin protects against oxidative stress-induced damage. For instance, Chiang et al. [63] demonstrated that fucoxanthin can protect heart valve interstitial cells against hydrogen peroxide-induced calcification, underscoring its potential therapeutic benefits for cardiovascular health. Similarly, Heo et al. [64] reported that fucoxanthin substantially enhanced the viability of Vero cells subjected to hydrogen peroxide-induced oxidative damage in a dose-dependent manner. Together, these findings support that while fucoxanthin can undergo partial degradation under elevated H_2_O_2_ exposure, it also functions as an effective antioxidant under physiological or low oxidative stress conditions.

Consistent with our findings, Baek et al. [65] also reported that fucoxanthin remains stable at temperatures up to 75 °C when ethanol is used as a solvent. The absence of changes in the UV-Vis spectra at different temperatures also suggests that no structural alterations occurred in the chromophore during thermal stability testing. Such structural integrity may be advantageous for specific applications in which the pigment’s color and properties must be preserved under moderate heat. These findings align with earlier studies on the heat stability of fucoxanthin. Wibowo et al. [66] reported that the optimal temperature for maintaining the all-*trans* version of fucoxanthin in *P. australis* was 60 °C, with only a 5.3% loss. This finding corroborates the current study’s observation of a 5% reduction at 60 °C within 5 h. Furthermore, Wibowo et al. [66] also highlighted that higher temperatures, such as 80 °C, trigger transformations to cis-isomers, indicating fucoxanthin’s thermal sensitivity. However, in this study, the loss of fucoxanthin was not accompanied by the appearance of isomers or degradation products, as previously reported [66]. Thus, the results of the present study show that short-term exposure to 60–70 °C during the preparation of skincare emulsions is unlikely to cause significant degradation of fucoxanthin, especially if the heating time is minimal and the formulation includes stabilizing agents.

The pH stability of fucoxanthin is crucial to its potential applications across industries, including pharmaceuticals, cosmetics, and functional foods. The results suggest that pH has a more pronounced detrimental effect on fucoxanthin stability than temperature does. Previous studies have reported that fucoxanthin is stable over pH 5–7, depending on the medium in which it is dissolved [67,68,69]. In this study, fucoxanthin dissolved in ethanol remained stable at pH 5. It was degraded at both acidic and alkaline extremes, underscoring the importance of controlling pH levels to preserve the integrity and efficacy of fucoxanthin-containing products. Since most skincare formulations are at pH 5, incorporating fucoxanthin into these products should not pose stability issues. Further research is needed to elucidate the mechanisms underlying pH-induced degradation and to explore the influence of different skincare media components on fucoxanthin stability.

The effects of high-intensity artificial UV radiation on fucoxanthin indicate that prolonged exposure negatively impacts its stability. The emergence of two small peaks corresponding to *cis*-isomers in the HPLC chromatogram coincided with a decreased fucoxanthin content, suggesting that the initial degradation involves the conversion of the stable all-*trans* version into oxidizable cis-isomers. These observations are consistent with previous studies on the light stability of fucoxanthin. Piovan et al. [70] also reported the accelerated degradation of fucoxanthin upon prolonged exposure to UV radiation. Baek et al. [65] highlighted the influence of solvent systems and co-extracts on fucoxanthin stability under UVA radiation over 24 h. However, in the present study, fucoxanthin was pure and exposed to artificial UV radiation across all wavelengths at high intensities (UVA: 19.9 W/m^2^, UVB: 21.4 W/m^2^, and UVC: 16.84 W/m^2^), which likely explains the rapid degradation observed within three hours. However, naturally occurring UV radiation along the Kuwait coast is much lower (UVA: 15.2 W/m^2^; UVB: 0.4 W/m^2^) [30]. Therefore, the stability of fucoxanthin in sunscreen lotions is unlikely to be a concern.

## 4. Materials and Methods

### 4.1. Chemicals

α,α-diphenyl-β-picrylhydrazyl (DPPH), ascorbic acid, ethylenediaminetetraacetic acid (EDTA), butylated hydroxytoluene (BHT), tetrabutylammonium acetate (TBAA), 4-(2-Hydroxyethyl)piperazine-1-ethane-sulfonic acid (HEPES), 5,5′-dithiobis(2-nitrobenzoic acid) (DTNB), and fucoxanthin reference materials were obtained from Sigma-Aldrich (Steinheim, Germany). Matrix metalloproteinase 1 (MMP1), Matrix metalloproteinase 12 (MMP12), and its substrate Ac-PLG-[2-mercapto-4-methyl-pentanoyl]-LG-OC_2_H_5_ were purchased from Enzo Life Sciences (Farmingdale, NY, USA). Mushroom tyrosinase and 3,4-dihydroxy-L-phenylalanine (L-DOPA) were purchased from Sigma–Aldrich (St. Louis, MO, USA). All other reagents were of analytical grade and obtained from Merck Millipore (Carrigtwohill, Ireland).

### 4.2. Algal Materials

Ten species of brown seaweeds were collected from the intertidal areas of the Kuwait coast of the Arabian Gulf between September 2021 and June 2022. *Iyengaria stellata*, *Feldmannia mitchelliae*, *Padina gymnospora*, and *Colpomenia sinuosa* were collected from beaches near the Kuwait Tower (29°23′37.3″ N, 47°59′59.6″ E). *Canistrocarpus cervicornis* was collected from Ahua Island (29°22′46.0″ N 48°26′23.6″ E). *Sargassum aquifolium* and *Sargassum asperifolium* were collected from Miscan Island (29°13′16″ N, 48°26′18″ E). *Sargassum angustifolium* and *Sargassum oligocystum* were collected from Salwa (29 °16′56.8″ N 48°05′20.4″ E). *Sargassum boveanum* was collected from Fintas (29°10′26″ N, 48°07′16″ E). Among these seaweeds, *P. gymnospora* was prevalent in the summer months, and all others were collected between September and March. For each seaweed species, approximately 2 kg of fresh biomass was collected. Freshly harvested seaweeds were washed with seawater, and their holdfasts and epiphytes were removed. A voucher specimen of each seaweed species was kept at the Kuwait Institute for Scientific Research (KISR). Immediately thereafter, the rinsed seaweeds were stored in a freezer (−40 °C). The seaweed samples were freeze-dried for two days (Coolsafe, Scanvac, Denmark), ground to a fine powder (Kneiftec™, KN195, FOSS, Hillerød, Denmark), and passed through a 0.5 mm sieve. The powdered seaweeds were stored at −80 °C under vacuum-packed conditions.

### 4.3. Isolation and Purification of Fucoxanthin

The fucoxanthin was isolated by the method described by Habeebullah et al. [12]. The isolated fucoxanthin was purified twice in a silica gel column by elution with hexane: acetone (7:3). The green fractions were discarded, and only the dark red and yellowish fractions were collected and dried under nitrogen. Fucoxanthin was recrystallized with dichloromethane: hexane (1:3). Fucoxanthin crystals were stored at −80 °C. The yield of pure fucoxanthin was estimated gravimetrically for each seaweed species. Yield was calculated by the following equation

$$Fucoxanthin\;Yield\;(mg/g\;dry\;weight)\;=\;\frac{Weight\;of\;purified\;fucoxanthin\;(mg)}{Dry\;weight\;of\;seaweed\;used\;(g)}$$

### 4.4. Determination of Purity by HPLC

The purity of the isolated fucoxanthin was analyzed using an Agilent 1100 Series UHPLC system (Agilent Technologies, Palo Alto, CA, USA) equipped with a diode array detector (DAD). An ion-pairing method, adapted from Van Heukelem and Thomas [71], was used for the analysis of algal pigments. Chromatographic separation was achieved using a Zorbax Eclipse XDB-C8 column (150 × 4.6 mm, 3.5 µm particle size; Agilent Technologies, Santa Clara, CA, USA). Gradient elution was performed using 70% methanol containing 30% 28 mM tetra-butyl ammonium acetate (TBAA, pH 6.5) as mobile phase A and methanol as mobile phase B at a flow rate of 1 mL/min. The injection volume was 20 μL. An injector program mixed the sample buffer (90% 28 mM TBAA, pH 6.5, and 10% methanol) with the sample or standard in the sample loop, then equilibrated for 3 min before the injection. The oven temperature was 60 °C. Individual pigments were detected using a diode array detector at 436, 405, 450, and 620 nm. Pigment identification and quantification were performed by comparing the chromatographic peaks of the samples with those of the standards under identical conditions. Twenty-two pigments, including chlorophyll c_3_, chlorophyll c_2_, pheophorbide A, peridinine, fucoxanthin, neoxanthin, prasinoxanthin, violaxanthin, astaxanthin, diadinoxanthin, alloxanthin, diatoxanthin, zeaxanthin, canthaxanthin, chlorophyll-b, divinyl chlorophyll-a, pheophytin A, lutein, chlorophyll-a, α-carotene, lycopene, and β-carotene can be separated by this method. The different pigment standards were obtained from DHI, Denmark. The fucoxanthin standard (Sigma-Aldrich analytical standard, HPLC grade ≥ 95.0% purity; catalog no. 16337-5MG, batch no. BCBQ1635V) was prepared at concentrations of 10–100 µg/mL. The main peak in the HPLC chromatogram was collected for NMR analysis.

### 4.5. Screening of Isolated Fucoxanthin for Bioactivity

#### 4.5.1. Antioxidant Activity

The antioxidant activity of the isolated fucoxanthin was determined using four in vitro assays: DPPH radical-scavenging activity, iron-chelating activity, reducing power, and inhibition of lipid peroxidation in a liposome model system, according to the method of Farvin et al. [72], in a microplate reader (Varioscan™ Lux, Thermo Scientific, Vantaa, Finland). Fucoxanthin samples were tested at concentrations of 0.06, 0.125, 0.25, and 0.5 mg/mL. The synthetic antioxidant BHT was used as a positive control for DPPH radical scavenging and in the liposome model system. Ascorbic acid and EDTA were used as positive controls for reducing power and iron-chelating activity, respectively. All assays were performed in triplicate.

#### 4.5.2. UV-Protective Properties of Isolated Fucoxanthin on *E. coli* Model System

Fucoxanthin isolated from five seaweed species, namely *C. cervicornis*, *F. mitchelliae*, *S. angustifolium*, *P. gymnospora*, and *S. asperifolium*, was used in the UV-protection study. *Escherichia coli* ATCC 25922 was used as the strain in this study. *E. coli* was streaked onto Luria–Bertani (LB) agar plates and incubated at 37 °C for 24 h. A single colony was inoculated into LB broth and incubated at 37 °C for 24 h. This culture was further diluted to a final concentration of 10^7^ CFU/mL. The mixture was centrifuged at 3500 rpm for 10 min, and the resulting pellets were rinsed twice with sterile normal saline. The pellets collected after centrifugation were resuspended in the same volume of normal saline to obtain a final concentration of 10^7^ CFU/ml. Fucoxanthin (100 μL) isolated from different seaweeds was dissolved in dimethyl sulfoxide (DMSO): H_2_O (9:1) at a concentration of 0.5 mg/mL and was mixed with 100 μL of *E. coli* culture in sterile 48-well microplates in triplicate. Then the plates were subjected to a high-intensity broad-spectrum artificial UV stress by exposing them to UV radiation in the range of 250–400 nm (UVA: 20.65 W/m^2^, UVB: 24.65 W/m^2^, UVC: 15.07 W/m^2^) for 30 min. The experiment was done in a BS-04 UV/VIS irradiation chamber (Opsytec Dr. Gröbel GmbH, Ettlingen, Germany) at room temperature with an exposure height of 25 cm. The lamps used included UVC-TUVF17T8 (Philips, Hamburg, Germany), UVB-TL20W/01RS Narrow band (Philips, Germany) and UVA-F18T8BLB (Sankyo Denki, Hiratsuka, Japan). After the exposure, 100 μL of this solution was serially diluted to 10^1^ and 10^2^. From this, 0.5 mL was spread plated onto LB agar plates. The plates were incubated at 37 °C for 48 h, and the number of surviving colonies was counted. The colony-forming units (CFU) were calculated. Controls used in this study include the following. (1) positive control (100% survival): 100 µL of *E. coli* culture mixed with 100 µL of DMSO:H_2_O (9:1) without UV exposure. (2) negative control: 100 µL of *E. coli* culture mixed with 100 µL of DMSO:H_2_O (9:1) and exposed to UV radiation under the same conditions. (3) fucoxanthin control: 100 µL fucoxanthin mixed with *E. coli* without UV exposure. The results were expressed as log CFU/mL. We used high UV radiation, including UVC, for two reasons. If a compound can protect against high-intensity UV radiation, including the deleterious effects of UVC, it suggests strong photoprotective potential. Moreover, at lower UV doses, particularly UVA or UVB alone, *E. coli* can undergo photorepair upon exposure to visible light [73,74], potentially masking the true protective effect of fucoxanthin.

#### 4.5.3. Anti-Collagenase and Anti-Elastase Activities of Isolated Fucoxanthin

The anti-wrinkle properties of fucoxanthin from *C. cervicornis*, *F. mitchelliae*, *S. angustifolium*, *P. gymnospora*, and *S. asperifolium* were determined by the inhibition of collagenase and elastase. The collagenase inhibition assay was performed by inhibition of Matrix Metalloproteinase-1 (MMP-1, fibroblast collagenase), and elastase inhibition was performed by inhibition of Matrix Metalloproteinase-12 (MMP-12, fibroblast elastase) [75]. Both assays were performed using the colorimetric drug discovery kit (Enzo Biochem, Inc. Farmingdale, NY, USA) according to the manufacturer’s instructions. Fucoxanthin was dissolved in DMSO:H_2_O (9:1, *v*/*v*). The assay was performed in 50 mM HEPES buffer (pH 7.5), containing 10 mM CaCl_2_, 0.05% Brij-35, and 1 mM DTNB. MMP-1 was diluted in the assay buffer to a concentration of 765 mU/μL, and 20 μL was added to each well. Similarly, MMP-12 was diluted to 35 mU/μL, and 20 μL was used per well. The mixture was preheated to 37 °C shortly before the assay. The synthetic chromogenic substrate (Ac-PLG-[2-mercapto-4-methyl-pentanoyl]-LG-OC_2_H_5_) was also prepared at a concentration of 1 mM and preheated to 37 °C. Twenty microliters of the samples (0.5 mg/mL) were incubated with 20 μL of MMP-1 or MMP-12 and 50 μL of buffer in a microplate well for 30 min at 37 °C to allow inhibitor enzyme interactions. Subsequently, 10 μL of the substrate was added, and the absorbance at 412 nm was continuously recorded using a microplate reader (Varioskan™ LUX, Thermo Scientific, Vantaa, Finland) at one-minute intervals for 10 to 60 min. A control was performed using only MMP and substrate without any inhibitor, and positive controls were performed using 6.5 μM of N-hydroxy-2-[[(4-methoxyphenyl)sulfonyl](2-methylpropyl)amino]-acetamide (NNGH). The experiments were performed in triplicate. The OD was plotted against time, and the slopes of the inhibitor and control were calculated. The percentage of inhibitor activity was calculated as follows:

% Inhibitor activity remaining = (slope of inhibitor/slope of control) × 100.

The % of collagenase inhibition = 100 − % inhibitor activity remaining.

#### 4.5.4. Tyrosinase Inhibition Activity of Isolated Fucoxanthin

Skin-whitening properties of fucoxanthin were determined by tyrosinase inhibitory activity. Mushroom tyrosinase was used to evaluate inhibitory activity using an established protocol with minor modifications [75]. Briefly, 50 µL of 0.5 mg/mL concentrations of each fucoxanthin, 80 μL of 50 mM phosphate-buffered saline (pH 6.5), and 20 μL of mushroom tyrosinase (1000 units/mL) in a 50 mM phosphate-buffered saline (pH 6.5) were added to a 96-well microplate (Corning, NY, USA). The reaction mixtures were then incubated at 37 °C for 30 min. Then, 50 μL of 1 mM L-DOPA was added to the mixture by multichannel pipette, and the amount of dopachrome produced in each well was measured at 492 nm using a microplate reader within 2 min. 0.75 mM of kojic acid was used as a positive control in a similar manner. The assay was performed in triplicate.

Tyrosinase inhibition rate (%) was calculated as follows:

% inhibition = 1 − (Absorbance of sample/Absorbance of control) × 100

### 4.6. NMR Analysis of Isolated Fucoxanthin

Structural analysis was conducted on nine of the 10 fucoxanthin samples. *Sargassum asperifolium* was excluded due to its unavailability in sufficient quantities. The samples were dissolved in deuterated methanol (MeOD). The ^1^H NMR spectra were acquired on a 600 MHz Bruker instrument (Bruker, Karlsruhe, Germany) operating at a frequency of 600.574 MHz for the proton channel. The zg30 pulse sequence was utilized from the pulse sequence library of Bruker TopSpin software 3.5. The recycle delay was 1s, and each spectrum was acquired with 128 repetitions. Moreover, representative HSQC and HMBC spectra of the species *C. cervicornis* are presented in the Supplementary Information. The pulse sequence hsqcetgpsp, already available in the Bruker library, which performs 2D ^1^H–^13^C correlation via double inept transfer with sensitivity improvement, was used to record HSQC spectra. For the acquisition of HMBC spectra, the pulse sequence hmbcgplpndqf, already available in the Bruker library and which generates 2D ^1^H–^13^C correlations via heteronuclear zero- and double-quantum coherence, was used. For the HSQC and HMBC experiments, the spectral widths were 8.00 ppm for the ^1^H dimension and 210.0 ppm for the ^13^C dimension. For the HSQC experiment, 128 transients were used in the indirect dimension (TD1), with 16 scans recorded per transient. For the HMBC experiment, the number of transients in the indirect dimension (TD1) was 128, with 32 scans recorded per transient. In the HSQC and HMBC measurements, the recycle delay times were set to 1.51 and 1.55 s, respectively.

### 4.7. Evaluation of the Stability of Isolated Fucoxanthin

The stability of isolated pure fucoxanthin was investigated under four different abiotic stress conditions: temperature, pH, artificial UV radiation, and the strong oxidizing agent H_2_O_2_. One mL of fucoxanthin isolated from *S. angustifolium* (0.25 mg/mL in ethanol) was dispensed into several 2 mL amber vials. For temperature stability, the samples were maintained at room temperature (20 °C) (0, 24, 48, and 72 h), 60 °C (0, 30 min, 1, 2, 3, 4, and 5 h), and 70 °C (0, 30 min, 1, 2, 3, 4, and 5 h). The pH stability of fucoxanthin was investigated by adjusting the pH to 4, 5, 6, 7, up to 11. Three vials were collected at each time point, and the UV-Vis spectra were measured with a microplate reader (Varioskan™ LUX, Thermo Scientific, Vantaa, Finland). The contents were analyzed by UHPLC (Agilent Technologies, Palo Alto, CA, USA). For the UVA + UVB + UVC stability studies, fucoxanthin was exposed to high UV radiation at different intervals (10 min, 30 min, 1 h, 2 h, and 4 h) in a 48-well microplate. The radiation doses used were UVA of 19.9 W/m^2^, UVB of 21.4 W/m^2^, and UVC of 16.84 W/m^2^ in an irradiation chamber (BS-04 UV/VIS irradiation chamber, Opstytec Dr. Globel, Ettlingen, Germany). At each time point, samples were collected from triplicate wells, UV-Vis spectra were obtained, and the contents were analyzed by UHPLC. The strong oxidizing agent H_2_O_2_ was tested at concentrations of 0%, 0.5%, 1%, and 2%.

### 4.8. Data Analysis

Results are expressed as mean ± SD of triplicate analyses. One-way analysis of variance (ANOVA) was performed, and statistical comparisons among groups were performed using Tukey’s multiple comparison test. Statistical significance was set at *p* <0.05. All data were analyzed using GraphPad Prism 4 (GraphPad Software, Inc., San Diego, CA, USA). NMR spectra were acquired using TopSpin 3.5, software provided by Bruker (Karlsruhe, Germany).

## 5. Conclusions

The findings of this study revealed significant species-specific variations in fucoxanthin yield and bioactive properties. The highest yield and UV-protective properties were observed for fucoxanthin from *P. gymnospora,* a brown seaweed that thrives in the extreme summer conditions of Kuwait, highlighting the ecological significance of these carotenoids in algal survival. The difference in the bioactivity of fucoxanthin isolated from different species stems from structural differences in the R group of fucoxanthin molecules. Fucoxanthin variants with the R group of CH_2_OCO(CH_2_)_4_CH_3_ (hexanoyloxyfucoxanthin) and CH_2_OCO(CH_2_)_2_CH_3_ (butanoyloxyfucoxanthin) exhibited bioactivity, with the former being more active. Fucoxanthin is stable under various conditions, including oxidizing agents and thermal stress. However, it is susceptible to high-intensity artificial UV radiation and sensitive to pH variations above 5. Most skincare formulations are at pH 5; therefore, incorporating fucoxanthin into these products should not pose any stability issues. These findings suggest the potential of fucoxanthin as a natural compound for UV protection, reducing oxidative stress, and potential for cosmetic applications. This study also underscores the importance of careful species selection for practical applications, as the bioactivity of fucoxanthin varies among species. Fucoxanthin variants from *P. gymnospora*, *C. cervicornis*, *F. mitchelliae*, and *S. angustifolium* are particularly promising, as they showed UV-associated cytoprotective activity in an *E. coli* model.

A limitation of this study is that UV radiation and other environmental parameters were not systematically monitored during sampling; therefore, their individual contributions to the observed variations cannot be determined. Further research is needed to clarify the mechanisms underlying the observed differences in structure and activity and to evaluate combinations of fucoxanthin and its variants from different species for enhanced UV-associated cytoprotection and potential application in skincare emulsions.

## Figures and Tables

**Table 1:** NMR assignments for the fucoxanthin from different brown seaweeds.

*C. sinuosa*		*I. stellata*		*S. aquifolium*		*S. angustifolium*		*P. gymnospora*		*S. boveanum*		*F. mitchelliae*		*S. oligocystum*		*C. cervicornis*	
NMR Chemical Shifts in ppm	Assignment of Proton Number	NMR Chemical Shifts in ppm	Assignment of Proton Number	NMR Chemical Shifts in ppm	Assignment of Proton Number	NMR Chemical Shifts in ppm	Assignment of Proton Number	NMR Chemical Shifts in ppm	Assignment of Proton Number	NMR Chemical Shifts in ppm	Assignment of Proton Number	NMR Chemical Shifts in ppm	Assignment of Proton Number	NMR Chemical Shifts in ppm	Assignment of Proton Number	NMR Chemical Shifts in ppm	Assignment of Proton Number
0.90	R	0.90	R	0.90	R	0.90	R	0.90	R	0.90	R	0.90	R	0.88		0.90	R
0.93	H13eq	0.93	H13eq	0.93	H13eq	0.93	H13eq	0.93	H13eq	0.93	H13eq	0.93	H13eq	0.90	R = CH_3_	0.93	H13eq
1.02	H14ax	1.02	H14ax	1.02	H14ax	1.02	H14ax	1.02	H14ax	1.02	H14ax	1.02	H14ax	0.93	H13eq	1.02	H14ax
1.19	H11	1.20	H11	1.07	R	1.19	H11	1.07	R	1.07	R	1.07	R	1.02	H14ax	1.07	
1.26	R	1.28	R	1.17	R	1.30	H42	1.18	R	1.18	R	1.18	R	1.07	R	1.19	H11
1.28	R	1.29	R	1.18	H11	1.32	R	1.19	H11	1.19	H11	1.19	H11	1.18	R	1.28	
1.32	H42	1.30	H42	1.28		1.38	H38ax	1.25		1.25		1.28		1.19	H11	1.30	H42
1.36	H6ax	1.40	H38ax	1.30	H42	1.45	R	1.30	H42	1.30	H42	1.30	H42	1.28		1.49	R
1.40	H38ax	1.70		1.38	H38ax	1.70	R	1.38	H38ax	1.38	H38ax	1.49	R	1.30	H42	1.70	R
1.50	H6eq	1.80	H4ax	1.83	H4ax	1.83	H4ax	1.40		1.40		1.60	R	1.49	R	1.83	H4ax
1.70		1.90	H16	1.92	H16	1.92	H16	1.49	R	1.49	R	1.83	H4ax	1.60	R	1.92	H16
1.80	H4ax	2.02	H38eq	2.02	H38eq	2.02	H38eq	1.70	R	1.70	R	1.92	H16	1.70	R	2.02	H38eq
1.90	H16	2.30	H4eq	2.20		2.20		1.83	H4ax	1.83	H4ax	2.02	H38eq	2.02	H38eq	2.10	R
2.02	H38eq	2.55	H8	2.25	H4eq	2.25	H4eq	1.92	H16	1.92	H16	2.10	R	2.10	R	2.25	H4eq
2.20		2.80	R	2.60	H8	2.35	H4eq	2.02	H38eq	2.02	H38eq	2.25	H4eq	2.25	H4eq	2.35	H4eq
2.35	H4eq	3.31	R	2.70	R	2.60	H8	2.27	H4eq	2.20	R	2.35	H4eq	2.35	H4eq	2.60	
2.60	H8	3.65		3.31	R	3.31	R	2.59	H8	2.25	H4eq	2.61		2.55		2.80	R
2.80	R	3.80		3.65		3.60		3.31	R	2.35	H4eq	2.80	R	2.61		3.31	R
3.31	R	4.02	R	3.80		3.80		3.60		2.60		3.05		2.80	R	3.50	
3.65		4.58	H16 (cis)	4.02	R	4.58	H16 (cis)	3.80		2.82	R	3.29	R	3.31	R	3.65	
3.80		5.40		4.30	R	5.35		4.64		3.31	R	3.59		3.65		3.82	
4.02	R	5.80	R	4.58	H16 (cis)	6.08		5.35		3.60		3.70		3.82		4.02	R
4.58	H16 (cis)	6.08		5.35		6.16	H30	5.49		3.65		3.80		4.02	R	4.22	R
5.35		6.16	H30	5.48		6.30	H25	5.80	R	3.80		4.20	R	5.35		4.72	
6.10		6.30	H25	5.80	R	6.38	H28	6.08		4.02	R	4.45	R	5.80	R	5.35	
6.16	H30	6.38	H28	6.06		6.50		6.16	H30	4.10	R	5.05		6.08		5.80	R
6.30	H25	6.50		6.16	H30	6.68	H29	6.30	H25	5.35		5.35		6.16	H30	6.08	
6.38	H28	6.70	H29	6.32	H25	6.82	H24	6.38	H28	5.49		5.49		6.30	H25	6.16	H30
6.50		6.82	H24	6.38	H28	7.32	H17	6.50		5.80	R	5.85	R	6.38	H28	6.30	H25
6.70	H29	6.97		6.50		8.11		6.81	H24	6.08		6.08		6.50		6.38	H28
6.82	H24	7.32	H17	6.68	H29	8.52		6.95		6.16	H30	6.16	H30	6.68		6.50	R
6.97		8.10		6.82	H24	9.40–9.65	CHO	7.32	H17	6.30	H25	6.30	H25	6.81	H24	6.68	R
7.32	H17	8.42		6.97				7.95	R	6.38	H28	6.38	H28	6.95		6.81	H24
8.10		9.40–9.65	CHO	7.32	H17			8.09		6.50		6.50	R	7.32	H17	6.95	
8.52				7.60	R			9.40–9.65	CHO	6.68		6.68	R	8.09		7.32	H17
9.40–9.65	CHO			7.95	R					6.81	H24	6.81	H24			7.95	
				8.11						6.95		6.95				8.09	
				8.21						7.32	H17	7.32	H17			8.19	
				8.10						7.90		7.95				9.40–9.65	CHO
				8.23						8.09		8.09				0.90	R
				9.40–9.65	CHO					8.28		8.33				0.93	H13eq
										9.40–9.65	CHO	9.40–9.65	CHO			1.02	H14ax

## Data Availability

All data supporting this study are included in the manuscript and Supplementary Materials.

## Supplementary Materials

The following supporting information can be downloaded at: https://www.mdpi.com/article/10.3390/molecules31213878/s1, Figures S1–S11: NMR spectra of fucoxanthin from Different Seaweeds; Figure S12: Superimposed HPLC chromatograms of purified fucoxanthin isolated from different seaweeds and fucoxanthin standard (Sigma). Peaks correspond to: (1) *S. oligocystum*, (2) *F. mitchelliae*, (3) *S. angustifolium*, (4) *S. aquifolium*, (5) *C. sinuosa*, (6) *S. boveanum*, (7) *S. asperifolium*, (8) *I. stellata*, (9) *C. cervicornis*, (10) *P. gymnospora*, and (11) fucoxanthin standard. 3D signal overlay was performed using Agilent’s OpenLab software. The overlay is within the full absorbance range, with a 10% absorbance offset between signals; Table S1: Concentration dependence of fucoxanthin in DPPH assay; Table S2: Concentration dependence of fucoxanthin in iron-chelating activity; Table S3: The table showing the fucoxanthin content of different algae studied; Table S4: Summary of the species and the analyses performed.

## Abbreviations

The following abbreviations are used in this manuscript:

UV	Ultraviolet
NMR	Nuclear magnetic resonance
USD	US dollar
DPPH	α,α-diphenyl-β-picrylhydrazyl
BHT	Butylated hydroxytoluene
EDTA	Ethylenediaminetetraacetic acid
TBARS	Thiobarbituric acid-reactive substances
DMSO	Dimethyl sulfoxide
MMP	Matrix metalloproteinases
NNGH	N-Isobutyl-N-(4-methoxyphenylsulfonyl)glycyl hydroxamic acid
TBAA	Tetrabutylammonium acetate
L-DOPA	3,4-dihydroxy-L-phenylalanin
