# Supplementary material for: Fucoxanthin and Its Variants from Seaweeds of the Arabian Gulf: Insight into Bioactivity, UV-Protective Properties, Stability and Cosmetic Potential
Source: Molecules. 2026 Oct 27;31(21):3878. doi: 10.3390/molecules31213878 (PMC13764730; doi:10.3390/molecules31213878)
Supplement: Supplementary file 1 [file molecules-31-03878-s001.zip › molecules-4617081-supplementary.pdf]

Supplimentary materials (S). The NMR spectra of fucoxanthin from Different Seaweeds

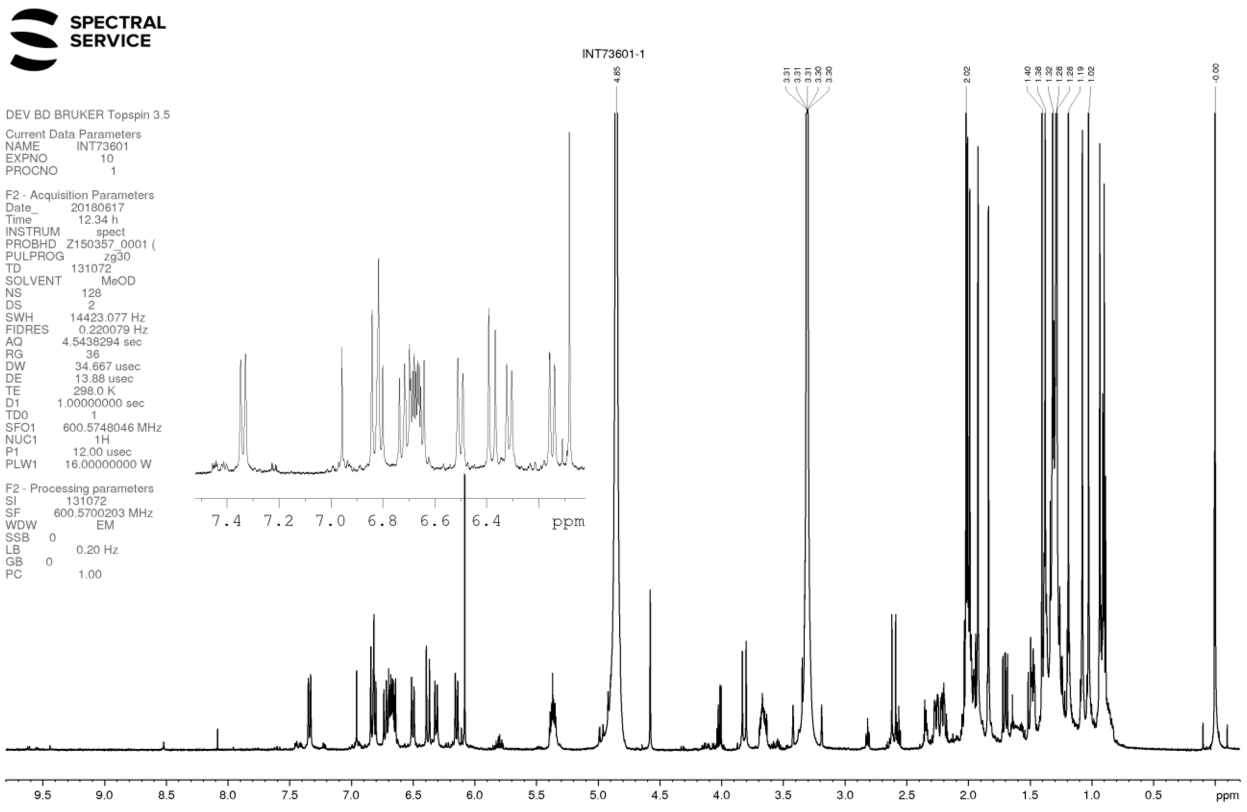

S1. NMR spectra of fucoxanthin from *Colpomenia sinuosa*

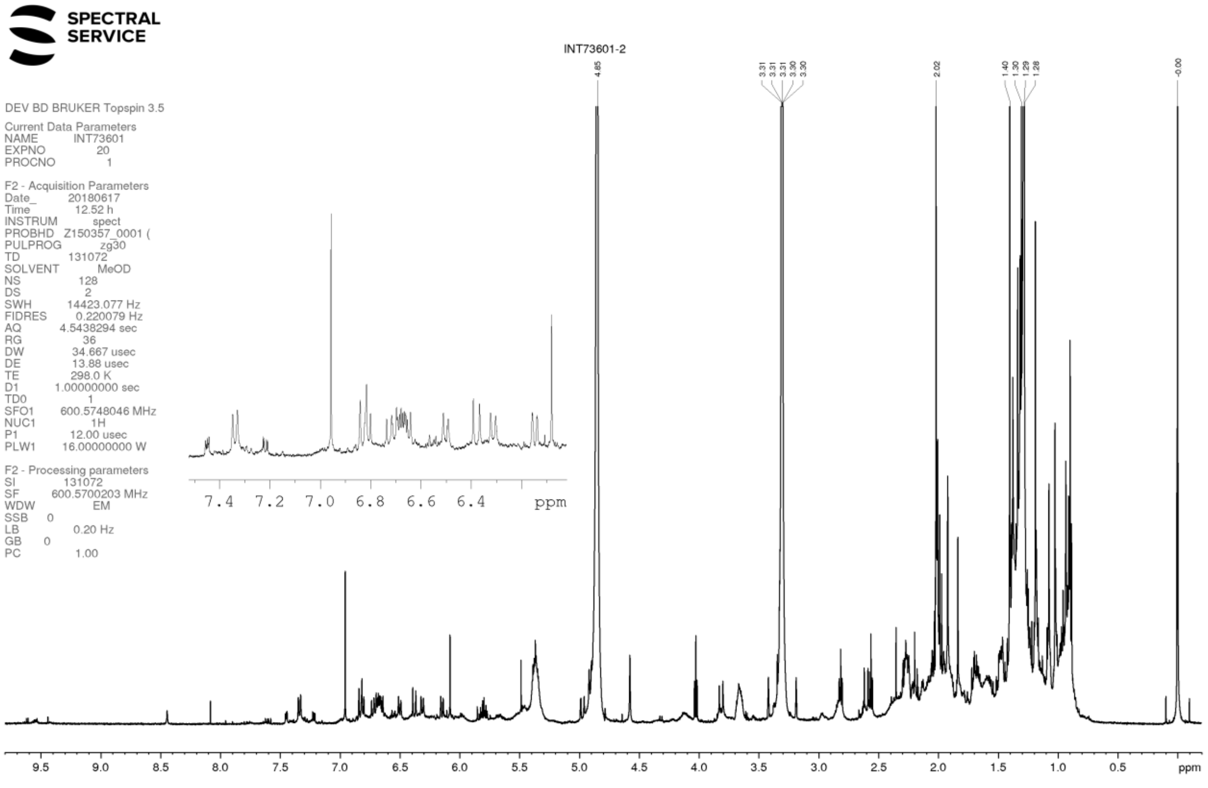

S2. NMR spectra of fucoxanthin from *Iyengaria stellata*

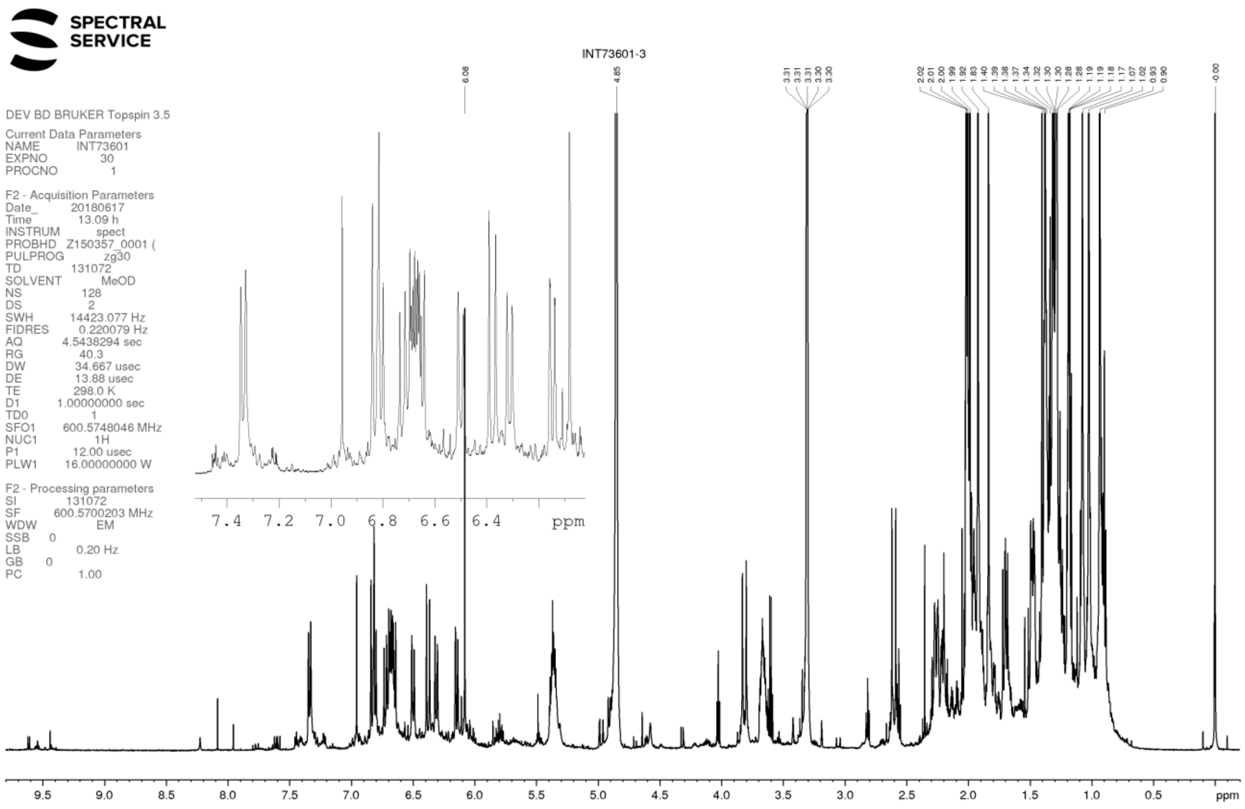

S3. NMR spectra of fucoxanthin from *Sargassum aquifolium*

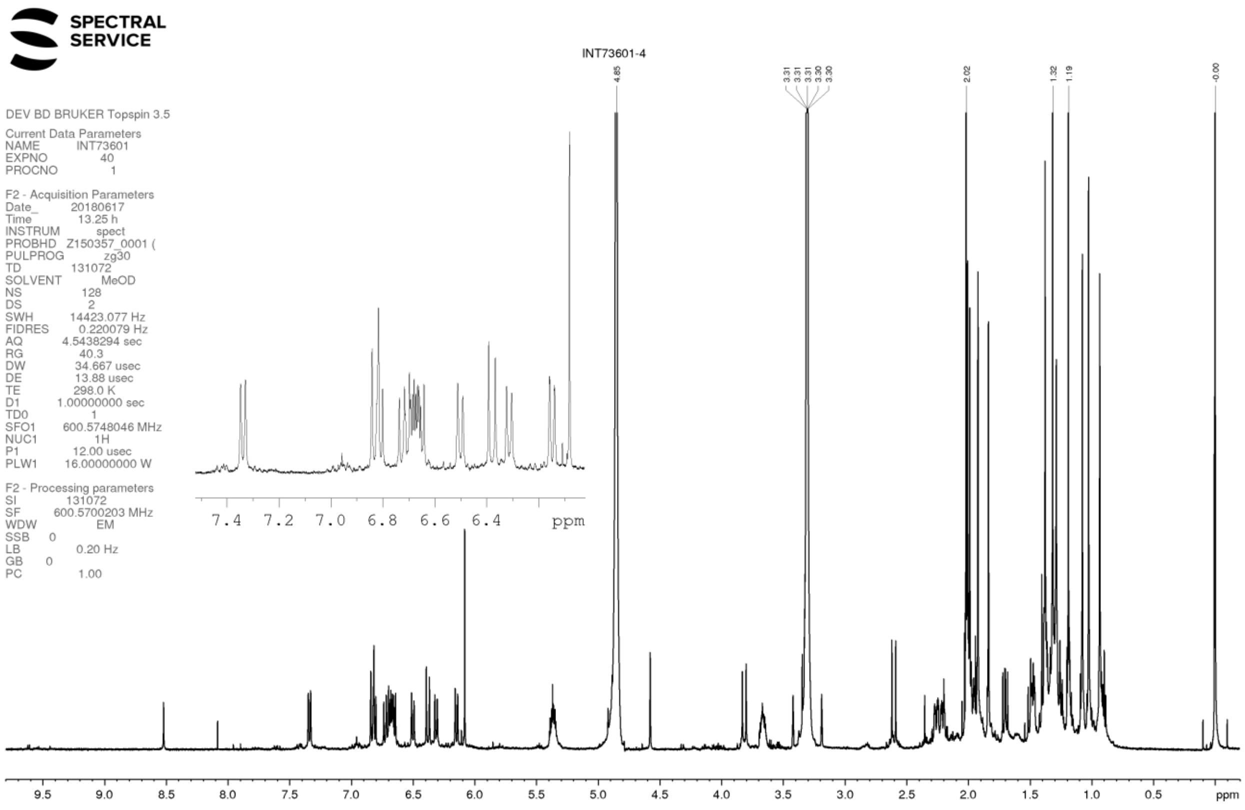

S4. NMR spectra of fucoxanthin from *Sargassum angustifolium*

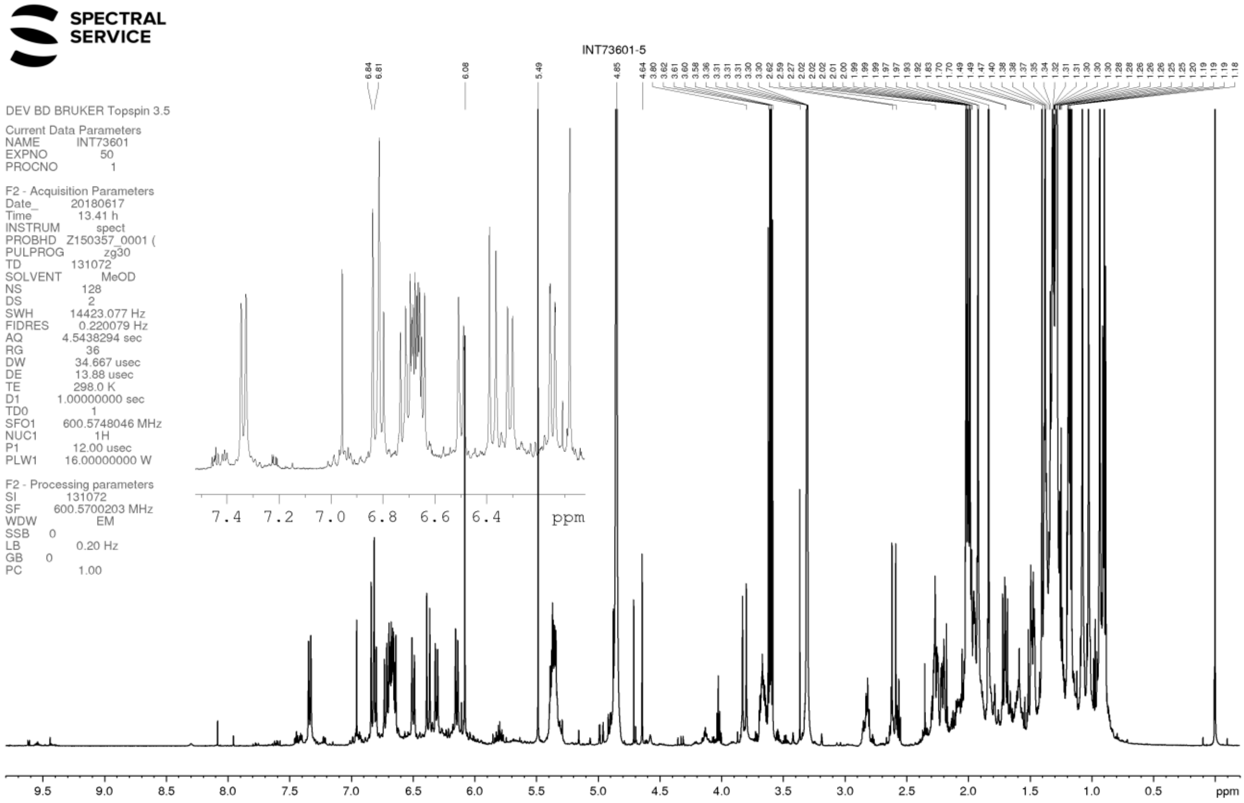

S5. NMR spectra of fucoxanthin from *Padina gymnospora*

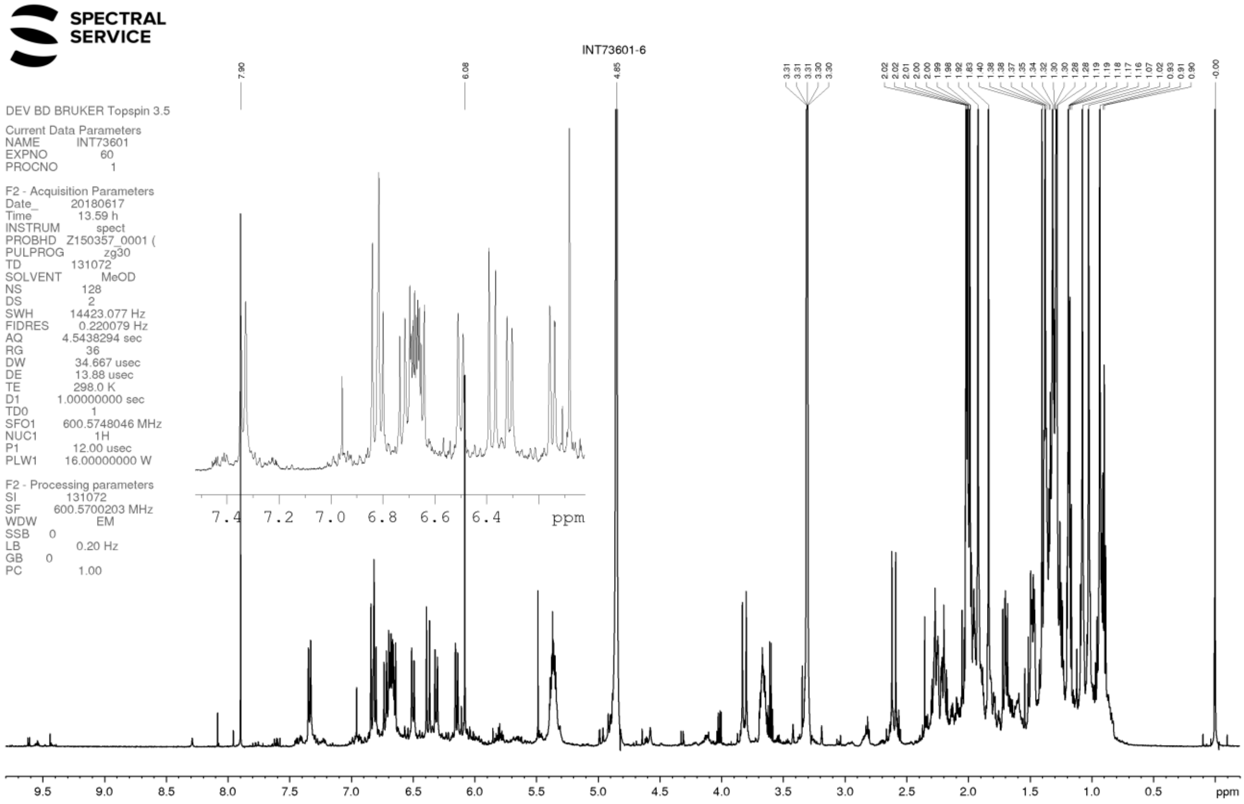

S6. NMR spectra of fucoxanthin from *Sargassum boveanum*

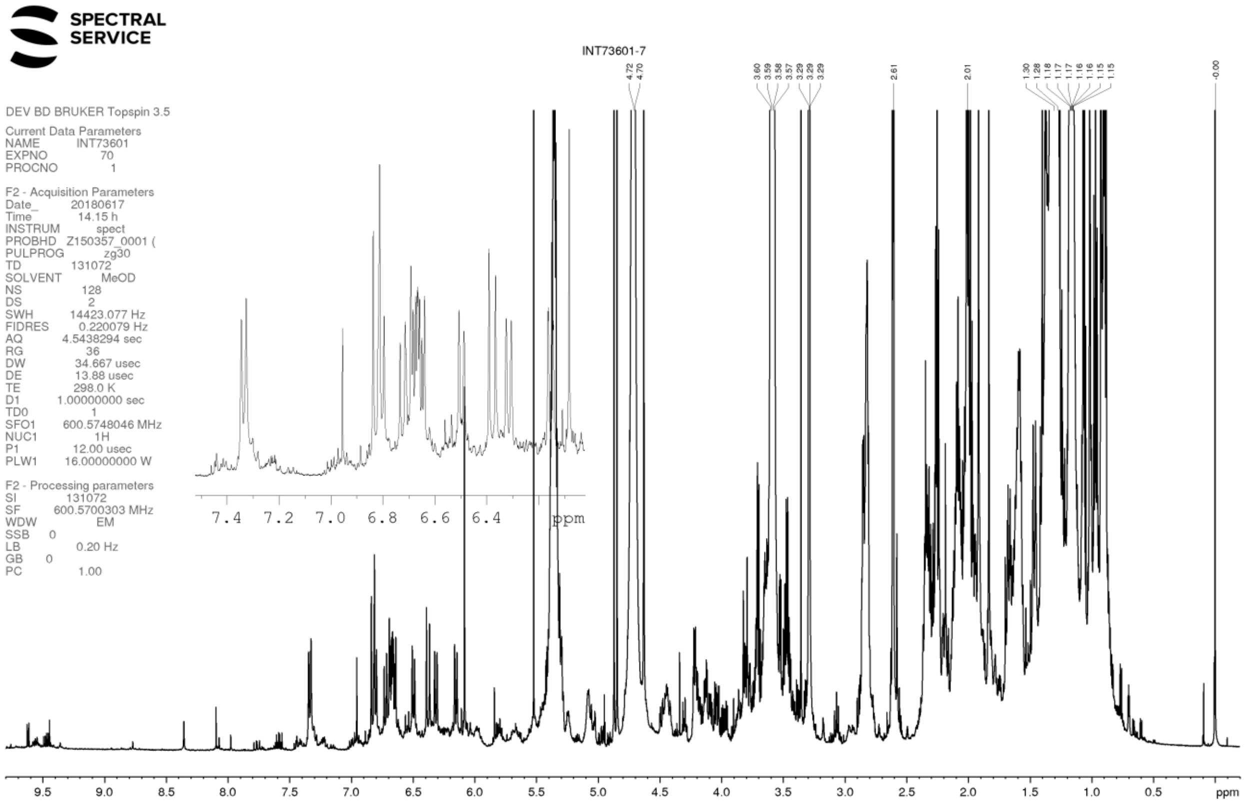

S7. NMR spectra of fucoxanthin from *Feldmannia mitchelliae*

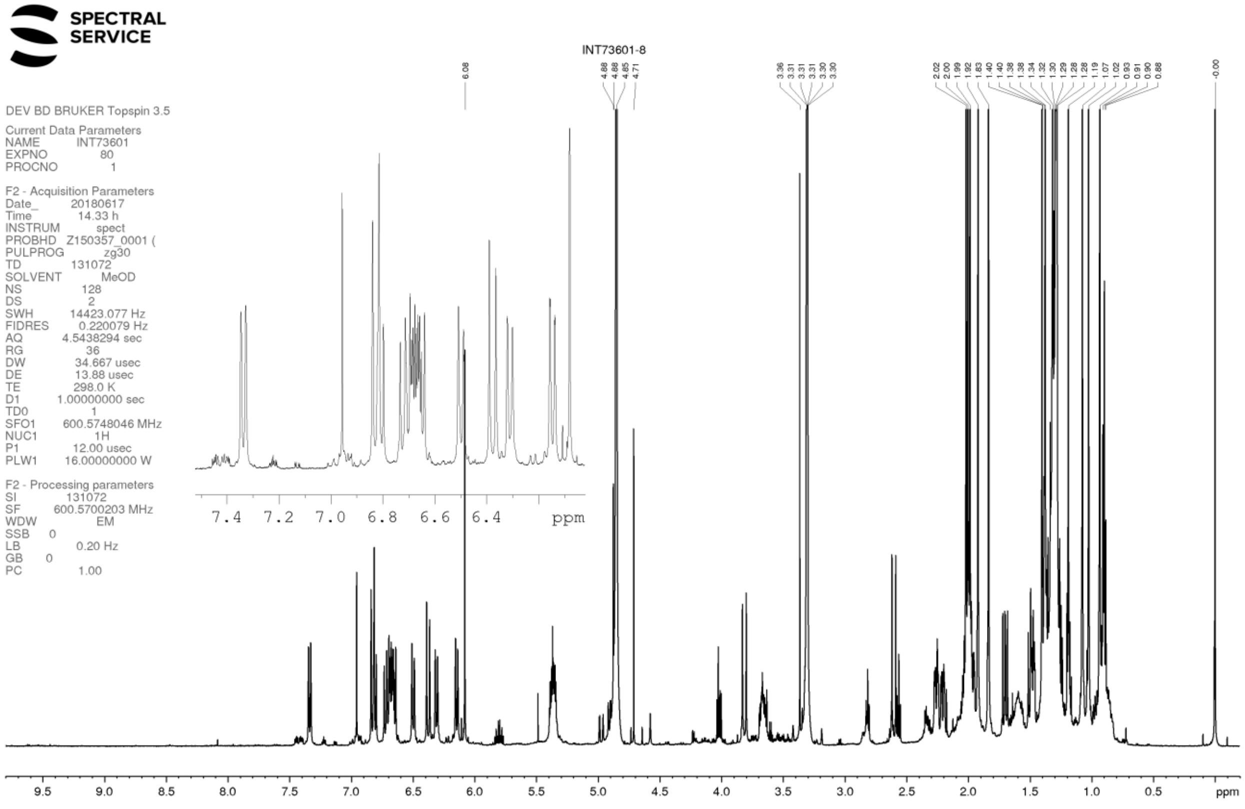

S8. NMR spectra of fucoxanthin from *Sargassum oligocystum*

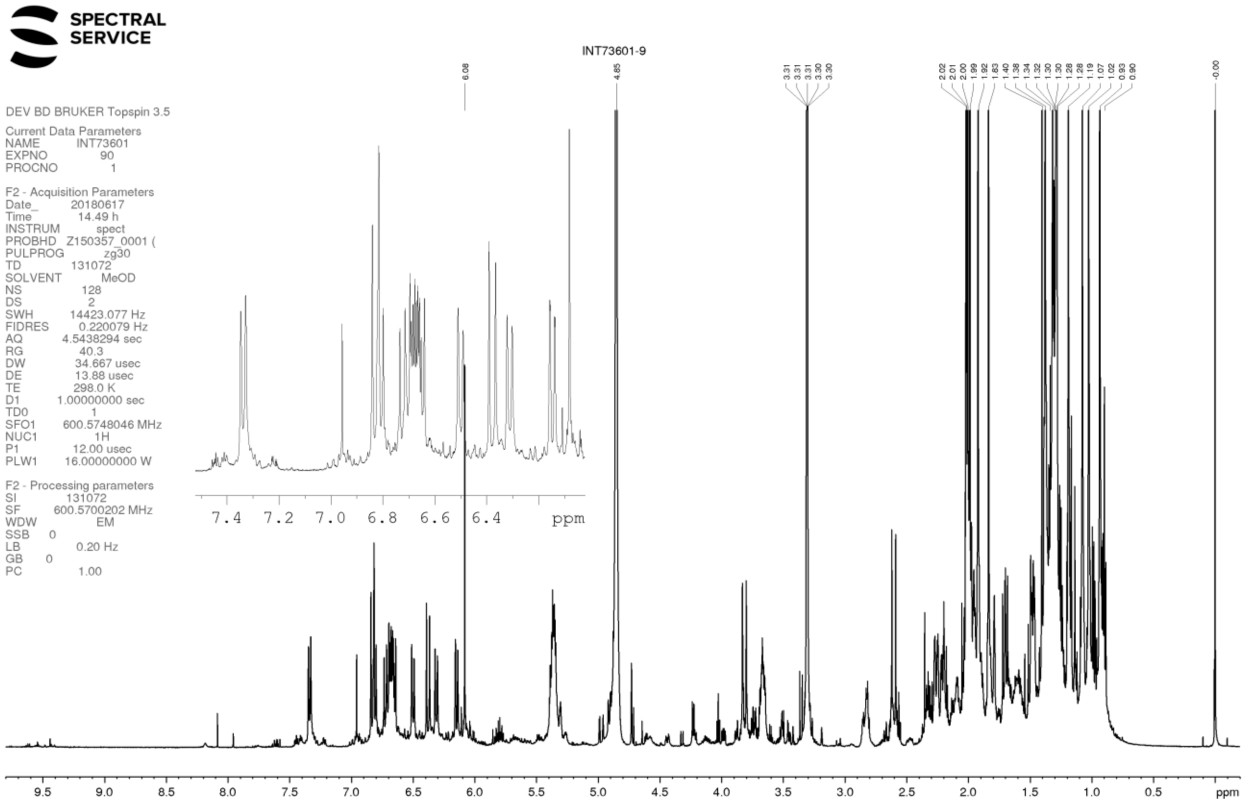

S9. NMR spectra of fucoxanthin from *Canistrocarpus cervicornis*

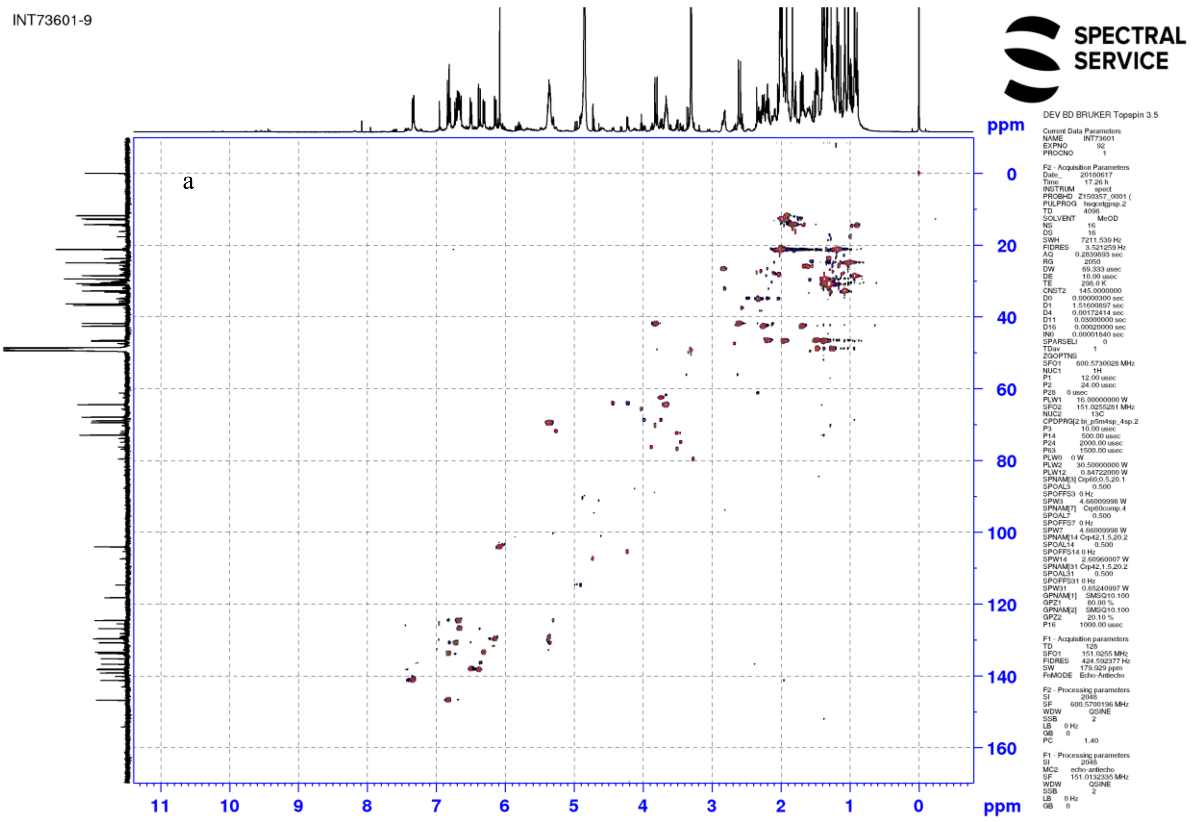
INT73601-9
SPECTRAL SERVICE
DEV BD BRUKER Topspin 3.5
a
ppm
ppm

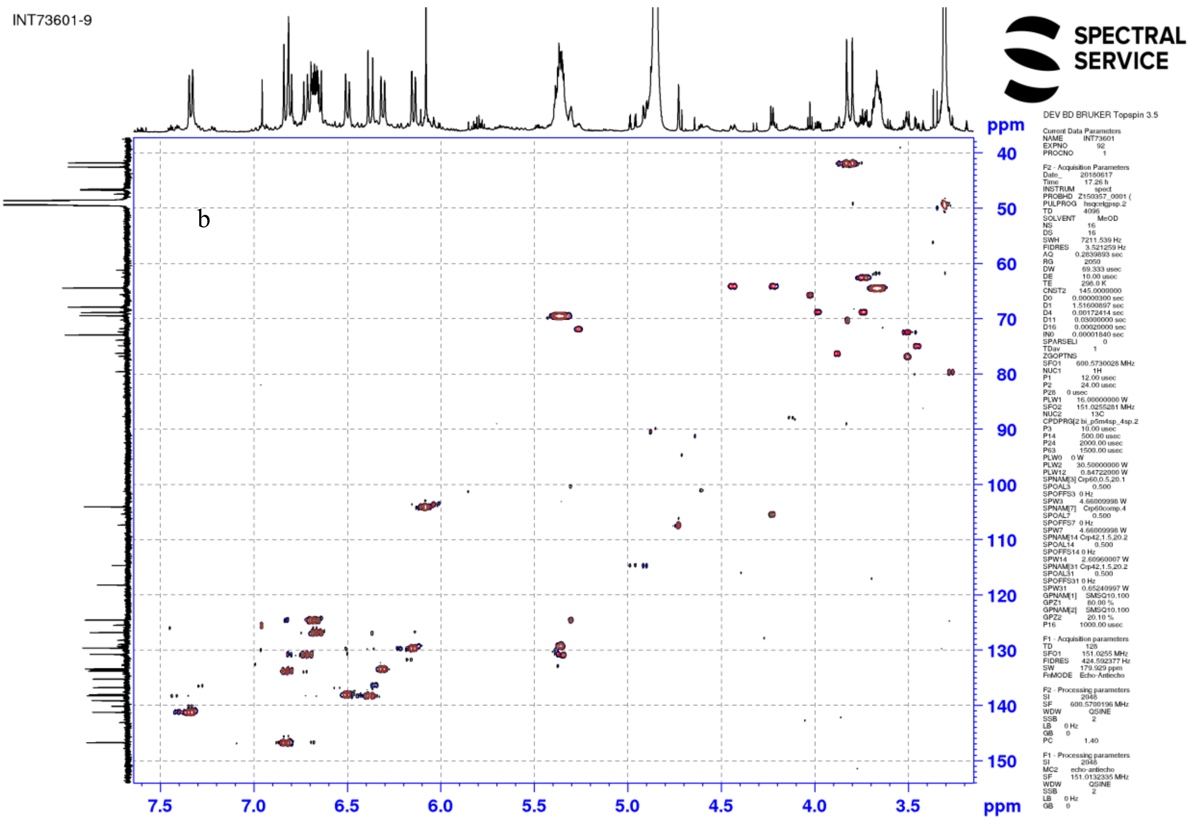
INT73601-9
SPECTRAL SERVICE
DEV BD BRUKER Topspin 3.5
b
ppm
ppm

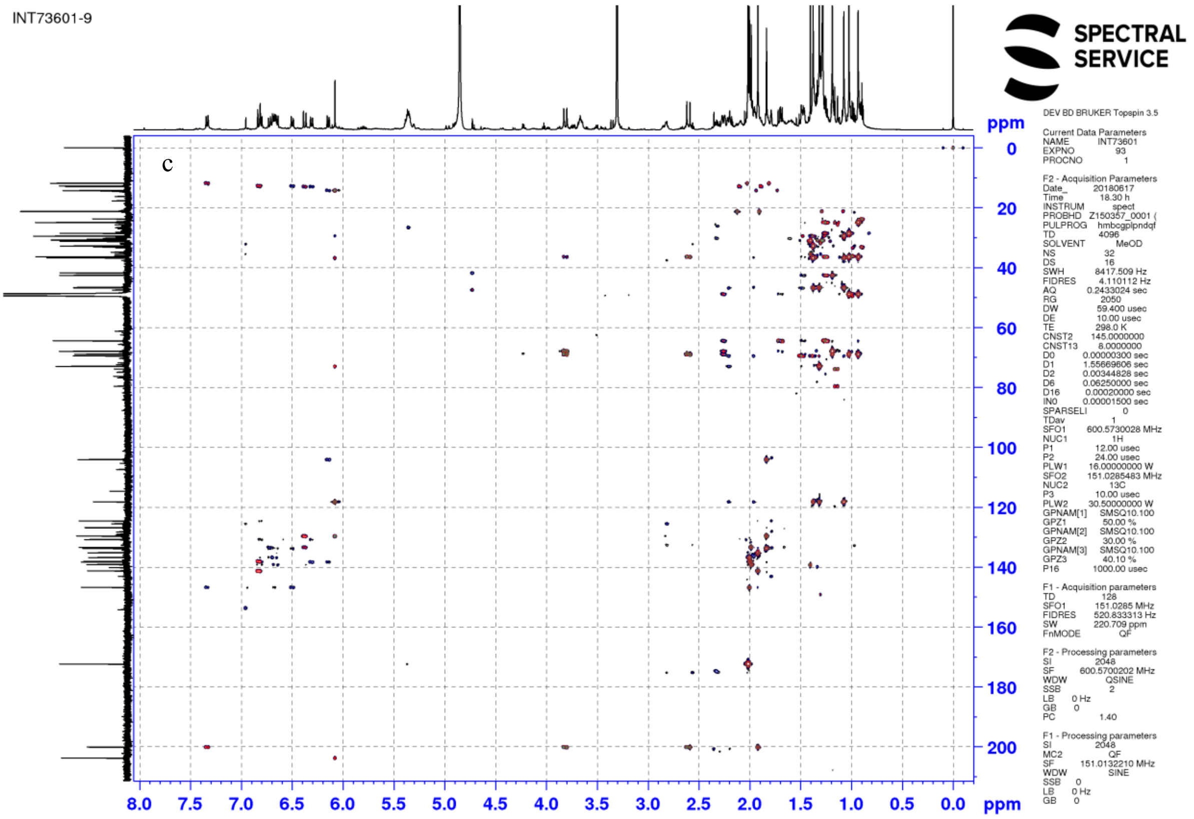

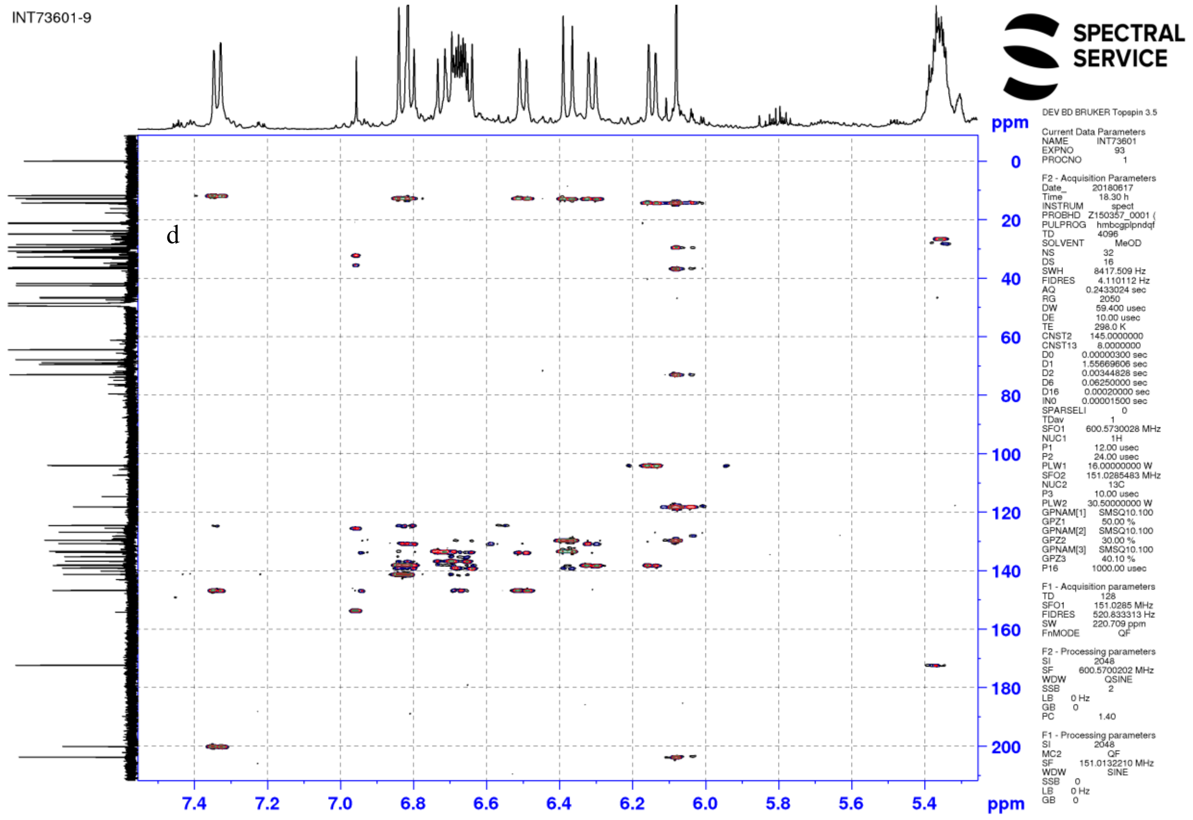

S10 a-d . 2 D NMR analysis of fucoxanthin

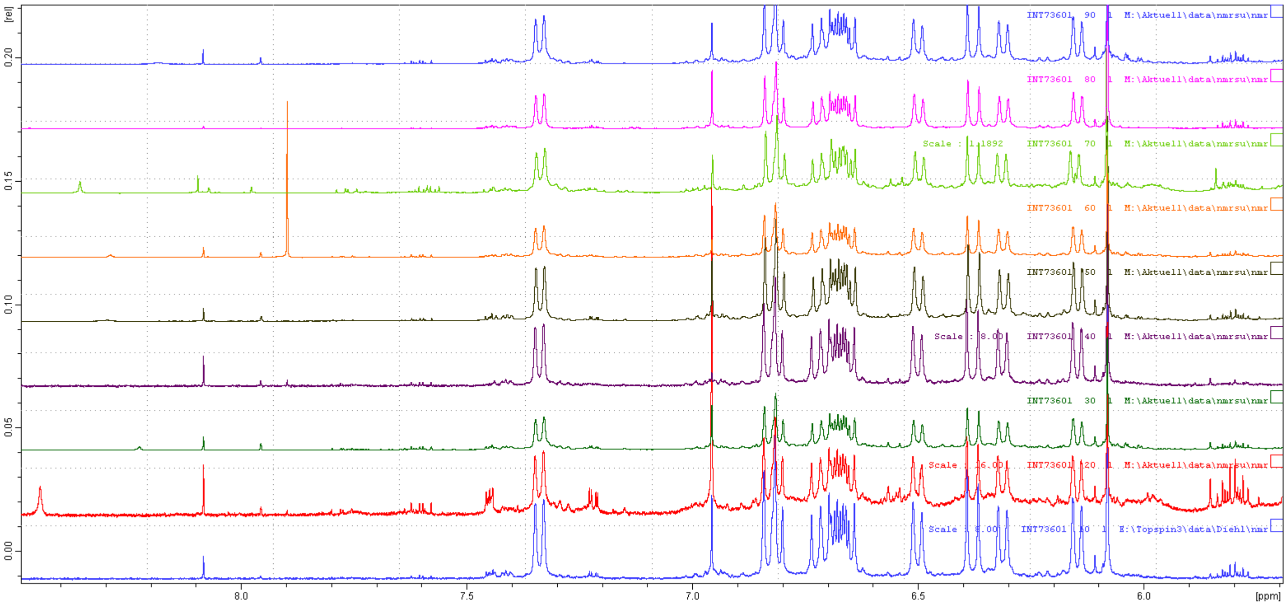

S11: NMR spectra of fucoxanthin showing the difference in their R groups

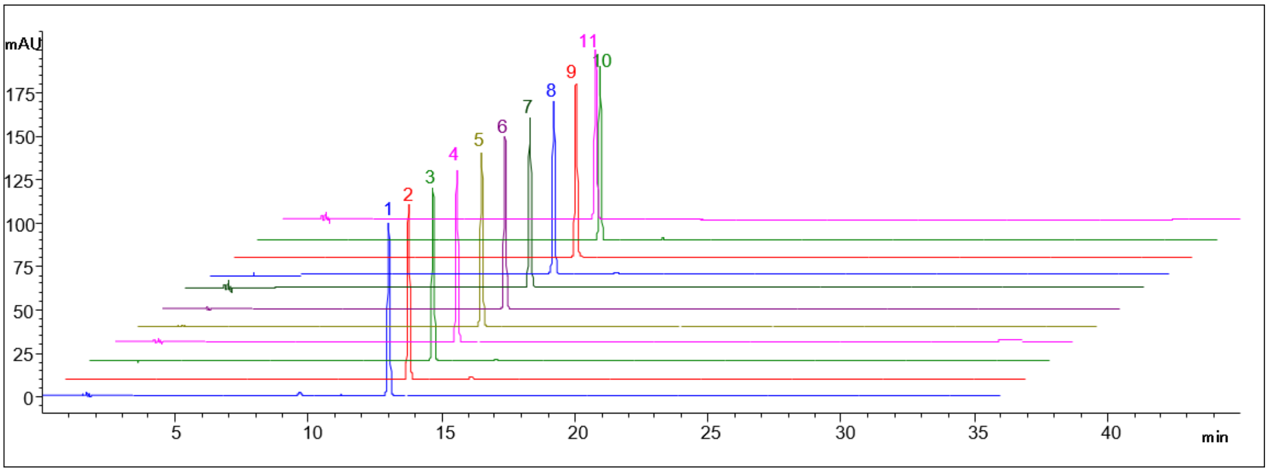

Figure S12. Superimposed HPLC chromatograms of purified fucoxanthin isolated from different seaweeds and fucoxanthin standard (Sigma). Peaks correspond to: (1) *S. oligocystum*, (2) *F. mitchelliae, (3) S. angustifolium*, (4) *S. aquifolium*, (5) *C. sinuosa*, (6) *S. boveanum*, (7) *S. asperifolium*, (8) *I. stellata*, (9) *C. cervicornis*, (10) *P. gymnospora*, and (11) fucoxanthin standard. 3D signal overlay was performed using Agilent's OpenLab software. The overlay is within the full absorbance range, with a 10% absorbance offset between signals.

Table S1. Concentration dependence of fucoxanthin in DPPH assay

	DPPH radical scavenging %			
**Concentration (mg/ml)**	**0.06**	**0.125**	**0.25**	**0.5**
I. stellata	35.2 ±2.7	41.0±4.4	57.6±6.8	81.7±2.2
F. mitchelliae	34.9±0.5	36.6±0.3	53.0±10.5	88.0±2.5
S. oligocystum	34.5 ±2.8	40.9±1.6	44.9±6.9	50.9±8.6
S. aquifolium	23.8±4.2	28.8±2.2	46.9±1.7	59.9±6.9
S. angustifolium	26.9± 5.9	36.5 ±4.7	57.5 ±2.6	77.0±8.9
C. sinuosa	32.6±1.3	39.8±1.5	54.0±3.7	60.9±2.5
C. cervicornis	57.0±7.9	64.9±6.2	86.9±1.0	92.7±2.3
P. gymnospora	32.8±4.1	37.6±2.4	47.6±4.5	67.6±1.5
S. boveanum	25.4±2.1	26.8±3.8	29.2±1.7	69.9±9.5
S. asperifolium	32.5±2.0	39.8±2.5	43.8 ±5.5	50.4 ±7.5

Table S2. Concentration dependence of fucoxanthin in iron chelating activity

	% Fe^{2+} Chelating activity			
**Concentration in mg/ml**	**0.06**	**0.125**	**0.25**	**0.5**
I. stellata	15.3±0.9	36.2±0.7	39.4±2.4	54.3±4.4
F. mitchelliae	30.4±4.3	32.0±5.2	39.2±5.03	48.6±3.5
S. oligocystum	29.3±0.8	31.9±3.1	39.9±1.6	50.3±5.8
S. aquifolium	27.5±1.5	34.4±4.9	44.3±1.2	48.4±4.3
S. angustifolium	26.4±0.9	30.2±0.8	35.5±2.2	45.7±5.9
C. sinuosa	25.9±1.5	30.9±1.0	39.4±1.3	47.5±3.9
C. cervicornis	35.6±2.4	42.3±2.3	49.3±1.	53.2±4.3
P. gymnospora	15.7±1.9	33.5±2.6	42.0±1.5	50.7±1.9
S. boveanum	16.3±1.1	30.1±2.8	48.4±1.8	52.9±3.3
S. asperifolium	25.0±2.3	33.0±3.6	40.0 ± 2.3	51.2±1.5

Table S3. The fucoxanthin content of different algae studied

Seaweed	Fucoxanthin content (mg/g)
I. stellata	0.80
F. mitchelliae	2.0
S. oligocystum	1.32
S. aquifolium	2.14
S. angustifolium	1.96
C. sinuosa	0.84
C. cervicornis	1.06
P. gymnospora	3.80
S. boveanum	2.72
S. asperifolium	0.71

Table S4. Summary of the species and the analyses performed

	Species	NMR analysis	Antioxidant activity	UV-protection studies	Collagenase inhibition	Elastase inhibition	Tyrosinase inhibition
1	*I. stellata*	Yes	Yes	—	—	—	—
2	*F. mitchelliae*	Yes	Yes	Yes	Yes	Yes	Yes
3	*S. oligocystum*	Yes	Yes	—	—	—	—
4	*S. aquifolium*	Yes	Yes	—	—	—	—
5	*S. angustifolium*	Yes	Yes	Yes	Yes	Yes	Yes
6	*C. sinuosa*	Yes	Yes	—	—	—	—
7	*C. cervicornis*	Yes	Yes	Yes	Yes	Yes	Yes
8	*P. gymnospora*	Yes	Yes	Yes	Yes	Yes	Yes
9	*S. boveanum*	Yes	Yes	—	—	—	—
10	*S. asperifolium*	—	Yes	Yes	Yes	Yes	Yes
